# Early Severe Inflammatory Responses to Uropathogenic *E. coli* Predispose to Chronic and Recurrent Urinary Tract Infection

**DOI:** 10.1371/journal.ppat.1001042

**Published:** 2010-08-12

**Authors:** Thomas J. Hannan, Indira U. Mysorekar, Chia S. Hung, Megan L. Isaacson-Schmid, Scott J. Hultgren

**Affiliations:** 1 Department of Molecular Microbiology and Center for Women's Infectious Disease Research, Washington University School of Medicine, St. Louis, Missouri, United States of America; 2 Department of Pathology and Immunology, Washington University School of Medicine, St. Louis, Missouri, United States of America; 3 Department of Obstetrics and Gynecology, Washington University School of Medicine, St. Louis, Missouri, United States of America; Yale University School of Medicine, United States of America

## Abstract

Chronic infections are an increasing problem due to the aging population and the increase in antibiotic resistant organisms. Therefore, understanding the host-pathogen interactions that result in chronic infection is of great importance. Here, we investigate the molecular basis of chronic bacterial cystitis. We establish that introduction of uropathogenic *E. coli* (UPEC) into the bladders of C3H mice results in two distinct disease outcomes: resolution of acute infection or development of chronic cystitis lasting months. The incidence of chronic cystitis is both host strain and infectious dose-dependent. Further, development of chronic cystitis is preceded by biomarkers of local and systemic acute inflammation at 24 hours post-infection, including severe pyuria and bladder inflammation with mucosal injury, and a distinct serum cytokine signature consisting of elevated IL-5, IL-6, G-CSF, and the IL-8 analog KC. Mice deficient in TLR4 signaling or lymphocytes lack these innate responses and are resistant, to varying degrees, to developing chronic cystitis. Treatment of C3H mice with the glucocorticoid anti-inflammatory drug dexamethasone prior to UPEC infection also suppresses the development of chronic cystitis. Finally, individuals with a history of chronic cystitis, lasting at least 14 days, are significantly more susceptible to redeveloping severe, chronic cystitis upon bacterial challenge. Thus, we have discovered that the development of chronic cystitis in C3H mice by UPEC is facilitated by severe acute inflammatory responses early in infection, which subsequently are predisposing to recurrent cystitis, an insidious problem in women. Overall, these results have significant implications for our understanding of how early host-pathogen interactions at the mucosal surface determines the fate of disease.

## Author Summary

The natural history of urinary tract infection (UTI) with uropathogenic *E. coli* in humans frequently includes persistent bacterial shedding in the urine for weeks after the initial infection, despite varying degrees of improvement of symptoms. Although antibiotic treatment has been very successful in treating UTI, antibiotic resistance is rising and recurrent infections are a common and costly problem affecting millions of women. Here, we examine an experimental mouse model of chronic bladder infection with uropathogenic *E. coli*, the most common cause of UTI. We found that the development of chronic bladder infection was preceded by severe bladder tissue inflammation that results in injury to the lining of the bladder. Furthermore, immunodeficient mice that lacked these acute inflammatory responses were protected from chronic bladder infection, suggesting that the development of chronic bladder infection requires immune-mediated tissue damage during acute infection. Finally, we demonstrate that mice with a history of chronic bladder infection that was subsequently cleared with antibiotic treatment are highly susceptible to further UTI. Thus, this study has discovered possible overlap in the mechanisms underlying the development of chronic and recurrent UTI.

## Introduction

Persistent microbial infections are a rapidly expanding problem because of increased antimicrobial resistance [1]. This trend is particularly concerning because of the increasingly appreciated role that chronic infections may play in cancer and chronic inflammatory diseases [2], [3], [4]. One key example is that of urinary tract infections (UTI), which are common, highly recurrent, and can become chronic [5], [6]. Females are disproportionately afflicted by UTI: 50% of all women will have an episode at some point in their lifetime, and 20 to 30% will have a recurrence within 3 to 4 months of the acute infection [7]. The high incidence of recurrent UTI (rUTI) suggests that many individuals do not develop protective immunity to uropathogens, though the capacity to do so has been demonstrated in both murine and primate experimental model systems and a phase 2 clinical vaccine trial in women [8], [9], [10]. This failure of adaptive immunity may be partially explained by host genetic and environmental factors, such as a maternal history of UTI and childhood exposure to uropathogens, which appear to play significant roles in determining susceptibility to rUTI [11], [12]. However, the molecular basis for rUTI and how this may relate to mechanisms of chronic infection, where adaptive immunity also falls short, are unknown. Furthermore, while antibiotic therapy has been resoundingly successful in treating acute UTI, recent increases in the prevalence of antibiotic-resistant uropathogenic strains in the community threaten to make chronic UTI common again [5], [13], [14]. Thus, understanding the host mechanisms contributing to chronic UTI, and other chronic bacterial infections of the mucosae, is of critical importance.

Uropathogenic *Escherichia coli* (UPEC) are by far the most common cause of UTI, accounting for 80% of outpatient infections and 25% of nosocomial infections [15]. During an acute episode, UPEC adhere to and invade the superficial facet cells of the urinary mucosal epithelium (urothelium) in a type 1 pili-dependent manner [16], [17]. UPEC invasion has been reported to involve several components of lipid rafts such as caveolin-1, an integral membrane protein found in the inner leaflet of the lipid bilayer [18]; Rac1, a member of the Rho family of GTPases [19], [20]; and microtubules [21]. After invasion, urothelial cells can expel UPEC via a TLR4-dependent exocytic pathway [22]. Alternatively, if UPEC escape into the cytoplasm, they can rapidly replicate, and subsequently aggregate into intracellular bacterial communities (IBC) [23]. Aggregation of UPEC into the biofilm-like IBC depends upon type 1 pili expression, independent of urothelial invasion, and is part of a mechanism for bacteria to evade extracellular host defenses while rapidly expanding in numbers during acute infection [24]. IBCs are transient in nature. Upon IBC maturation during approximately the first 12 to 16 hours of infection, the bacteria detach from the biomass and flux back into the lumen, spreading to neighboring epithelial cells where they are capable of initiating another IBC [25]. IBC formation has only been observed in the early acute stages of infection [25]. However, the ability of UPEC to expand in numbers via IBC formation has been shown to be a prerequisite for persistence as mutants that are defective in IBC formation are highly attenuated and rapidly cleared from the urinary tract [26]. IBC formation has been observed in multiple murine backgrounds with numerous UPEC strains [27]. Evidence of IBC formation has also been found in the bladders of mice infected with *Klebsiella pneumoniae* and in urine sediments from women with acute cystitis by UPEC, indicating that this intracellular pathogenic cycle is not unique to UPEC infection of mice [28], [29]. UPEC colonization and invasion of the urothelium triggers innate host responses, which are mediated in part by Toll-like receptor 4 (TLR4), a pattern recognition receptor that responds to certain pathogen-associated molecular patterns such as lipopolysaccharide [30], [31], [32]. These early innate responses include bacterial expulsion, urothelial exfoliation, and bladder inflammation that is characterized by the production of the pro-inflammatory cytokine interleukin 6 (IL-6), granulocyte chemotactic cytokines such as IL-8, the hormone granulocyte colony stimulating factor (G-CSF), and the T cell-associated, pro-inflammatory cytokine IL-17A [17], [33], [34], [35], [36], [37], [38].

In humans, the ultimate outcome of UPEC infection of the urinary tract ranges from acute, self-limiting infection to asymptomatic bacteriuria (presence of bacteria in the urine), to recurrent or chronic UTI [7]. Diminished TLR4 receptor expression and CXCR1 (IL-8 receptor) signaling have been associated with increased susceptibility to asymptomatic bacteriuria and severe pyelonephritis, respectively, in children, highlighting the importance of the innate immune response in determining disease outcome [39], [40]. However, the host mechanisms that contribute to acute, recurrent and chronic cystitis in adult women are poorly understood. While mast cells, γδ T cells, and neutrophils have each been implicated in facilitating clearance of acute UTI in C57BL/6J mice [34], [41], [42], [43], recent studies have suggested that the role of neutrophils may be more complex than previously appreciated, highlighting the need for further studies [33], [44]. Adding to this complexity is the fact that the outcome of experimental UPEC infection differs substantially between inbred mouse strains [45]. In C57BL/6J mice, which typically resolve acute infection and bacteriuria within 7–10 days, a small intracellular population of bacteria resistant to antibiotic therapy persists latently for months in the bladder urothelium of these mice [46], [47], [48], [49]. This quiescent intracellular reservoir (QIR) is distinct from the IBC as it is comprised of fewer than 15 bacteria persisting in a membrane bound dormant state [49]. QIRs are capable of re-emerging from dormancy to cause recurrences of infection and bacteriuria, and may represent one mechanism for rUTI in humans [49]. In contrast, some C3H background mouse strains have been reported to develop chronic UTI for up to 2 weeks post-infection [45], [50]. These latter strains may reflect in part the natural course of UTI in women, as placebo-controlled studies have demonstrated that a majority of women remain bacteriuric weeks after an acute UTI if not treated with antibiotics, despite overall improvement of symptoms [51], [52]. Thus, these murine models of chronic UTI, which are not well understood, merit further study as they could reveal critical host determinants of UTI pathogenesis.

In this study, we developed an experimental chronic infection model to study aspects of the host response that determine disease outcome. Using a C3H/HeN murine model of cystitis, we demonstrated that while acute, self-limiting infection occurs in a subset of mice, another potential outcome of UPEC infection is the establishment of chronic cystitis lasting months. We discovered acute biomarkers that are predictive for the development of chronic cystitis and showed that the development of chronic cystitis was dependent upon severe acute inflammation. Furthermore, results from challenge infections following antibiotic therapy indicated that a history of chronic cystitis was a significant risk factor for subsequent severe and chronic infections. These findings suggest that a common mechanism may underlie both chronic and recurrent bacterial infections.

## Results

### C3H/HeN mice develop chronic cystitis in response to UPEC infection in an infectious dose-dependent manner

**Figure 1:**
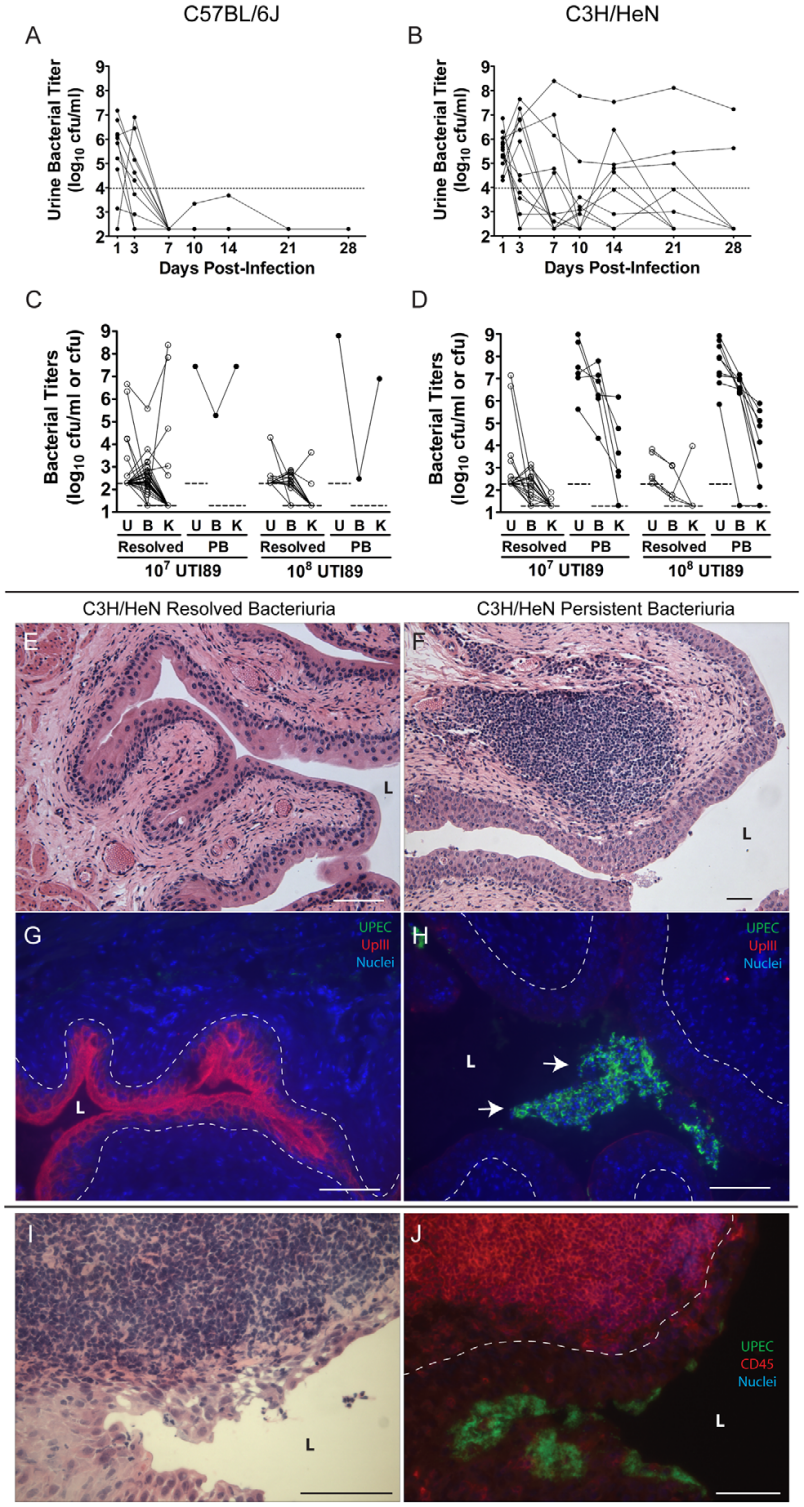
C3H/HeN mice develop chronic cystitis in response to UPEC infection in an infectious dose-dependent manner. *A–B*, Representative urine bacterial titer time course over 4 wpi with 10^7^ cfu UTI89 Kan^R^ in *A*, C57BL/6J and *B*, C3H/HeN mice. Solid lines connect the urine titers over time for each individual mouse. Dashed horizontal line represents the cutoff for significant bacteriuria in free catch urines: 10^4^ cfu/ml. *C–D*, Urine (**U**), bladder (**B**) and kidney (**K**) titers of *C*, C57BL/6J and *D*, C3H/HeN mice at 4 wpi with 10^7^ or 10^8^ cfu UTI89 Kan^R^ or UTI89, grouped by outcome: resolved bacteriuria (**Resolved**) or persistent bacteriuria (**PB**). Solid lines connect the different urine and tissue titers from the same mouse. Dotted horizontal lines show the limits of detection. *E–H*, Paraffin-embedded bladder sections 4 wpi with UTI89 Kan^R^ were examined by *E–F*, Hematoxylin & eosin (H&E) staining and light microscopy, or *G–H*, indirect immunofluorescence (IF) microscopy, staining with antibodies against *E. coli* (green) and uroplakin III (red); nuclei are counterstained with bis-benzimide (blue). Bladder sections from mice that resolved bacteriuria are displayed in panels *E* and *G*; from persistently bacteriuric mice in panels *F* and *H*. In panel *H*, *arrows* indicate luminal clusters of bacteria associated with immune and exfoliated urothelial cells. *I–J*, Frozen sections of the bladder of a persistently bacteriuric C3H/HeN mouse at 7.5 months post-infection with UTI89 were examined: *I*, H&E stained section of the trigone region (area of bladder near urethra where ureters enter), *J*, IF micrograph of a serial section of *I*, staining for CD45 (red) and the FimH adhesin of *E. coli* (green); nuclei are counterstained with bis-benzimide (blue). In panels *E–J*, *bars* approximate 50µm, “*L*” indicates bladder lumen, and *dashed line* denotes the approximate location of the urothelial basement membrane.

C57BL/6J mice typically resolve acute UPEC UTI by 2 weeks post-infection (wpi), with sterile urines and kidneys and low level bladder colonization (<10^4^ colony-forming units (cfu) UPEC per bladder) indicative of a latent QIR population [46]. In contrast, we previously demonstrated that a large percentage of C3H/HeN mice have high level bladder colonization (>10^4^ cfu UPEC per bladder) at 2 wpi [29]. However, C3H/HeN mice had not been previously reported to be susceptible to chronic UTI [32], [45]. To determine whether this finding represented an alternative disease outcome or merely a high rate of recurrence, we performed comparative long-term UPEC infection studies investigating the progression of UTI in C57BL/6J and C3H/HeN mice. Female mice were infected at 7–8 weeks of age with 10^7^ cfu of a kanamycin-resistant derivative of the UPEC strain UTI89, UTI89 Kan^R^, unless otherwise indicated, and then followed by longitudinal urinalysis over 4 wpi. We found that C3H/HeN mice were significantly more susceptible to developing persistent bacteriuria, as defined by the recovery of greater than 10^4^ cfu UTI89 per ml of urine at all time points over 4 wpi, compared to C57BL/6J mice (Table 1; Fig. 1A–B). Urine titers of 10^4^ cfu/ml or more had previously been demonstrated to be a reasonable cutoff for indicating the presence of urinary tract infection in mice when assaying free catch urine samples [46]. Increasing the inoculum to 10^8^ cfu UTI89 significantly increased the incidence of persistent bacteriuria in C3H/HeN mice from 21% to 52% (*P*<0.01, Fisher's Exact test), while C57BL/6J mice remained resistant to persistent bacteriuria (Table 1). Resolution of bacteriuria was not observed in C3H/HeN mice after 4 wpi in 12 persistently bacteriuric mice from two independent experiments that were followed for 6–8 months post-infection (data not shown). Therefore, in response to UPEC infection a subset of C3H/HeN mice develop persistent bacteriuria lasting months in an infectious dose-dependent manner.

**Table 1:** The incidence and severity of chronic UTI among inbred mouse strains.

Mouse Strain	UTI89 Kan^R^ InoculumCFU	Incidence of Persistent Bacteriuria^A^ % (ratio)	Bladder Titers				Kidney Titers	
			Persistent Bacteriuria^A^		Resolved Bacteriuria^B^		Persistent Bacteriuria^A^	Resolved Bacteriuria^B^
			Bladder Titers Mean of Log_10_ values ± SD (n)	PPV^C^ for >10^4^ Bladder CFU % (ratio)	Bladder Titers Mean of Log_10_ values ± SD (n)	NPV^C^ for >10^4^ Bladder CFU % (ratio)	Kidney Titers Mean of Log_10_ values ± SD (n)	Kidney Titers Mean of Log_10_ values ± SD (n)
C3H/HeN	10^7^	21 (17/82)^D^	6.4±1.2 (6)†	100 (6/6)	2.0±0.6 (27)	100 (27/27)	3.6±1.7 (6)†	1.3±0.1 (27)
	10^8^	52 (35/67)^D^	6.1±1.8 (9)†	89 (8/9)	2.2±0.7 (6)	100 (6/6)	3.9±1.6 (9)†	1.7±1.1 (6)
C57BL/6J	10^7^	5 (2/44)* D	5.3 (1)	100 (1/1)	2.3±0.9 (34)	97 (33/34)	7.4 (1)	1.9±1.7 (34)
	10^8^	7 (1/15)* D	2.5 (1)	0 (0/1)	2.3±0.5 (14)	100 (14/14)	6.9 (1)	1.5±0.7 (14)
C3H/HeOuJ	10^7^	92 (22/24)*	6.6±0.8 (22)†	100 (22/22)	1.8±0.2 (2)	100 (2/2)	6.1±1.0 (22)†	1.3±0.0 (2)
	10^8^	93 (13/14)^D^	7.2±0.4 (13)	100 (13/13)	5.3 (1)	0 (0/1)	6.7±1.5 (13)	1.9 (1)
C3H/HeJ	10^7^	80 (16/20)* E	4.3±2.1 (16)	44 (7/16)	2.7±0.6 (4)	100 (4/4)	6.0±1.3 (16)†	3.7±1.6 (4)
	10^8^	100 (4/4)^F^	6.2±2.5 (4)	75 (3/4)	N/A	N/A	6.5±1.6 (4)	N/A
C3H/HeSnJ	10^7^	40 (2/5)^D^	4.6±3.3 (2)	50 (1/2)	1.5±0.2 (3)	100 (3/3)	4.0±3.4 (2)	1.5±0.3 (3)
	10^8^	62 (16/26)^G^	7.1±0.5 (16)†	100 (16/16)	2.6±0.6 (10)	100 (10/10)	5.7±0.7 (16)†	1.6±0.5 (10)
C3HeB/FeJ	10^7^	20 (2/10)	6.6±0.2 (2)†	100 (2/2)	2.3±0.6 (8)	100 (8/8)	4.1±0.4 (2)†	1.6±0.5 (8)
	10^8^	44 (4/9)	7.0±0.4 (4)†	100 (4/4)	2.1±0.6 (5)	100 (5/5)	4.9±1.0 (4)†	1.4±0.1 (5)
CBA/J	10^7^	20 (2/10)	6.9±0.3 (2)†	100 (2/2)	2.1±0.4 (8)	100 (8/8)	5.5±2.3 (2)†	1.3±0.0 (8)
	10^8^	30 (3/10)	7.0±0.3 (3)†	100 (3/3)	2.7±1.8 (7)	86 (6/7)	6.9±0.3 (3)	2.1±2.2 (7)
DBA/2J	10^7^	40 (4/10)	5.3±2.2 (4)†	75 (3/4)	2.1±0.5 (6)	100 (6/6)	2.7±2.3 (4)	1.3±0.0 (6)
	10^8^	20 (2/10)	3.2±1.9 (2)	50 (1/2)	1.7±0.2 (8)	100 (7/7)	1.3±0.0 (2)	1.3±0.0 (8)
BALB/cJ	10^7^	0 (0/5)^D^	N/A	N/A	2.5±1.1 (5)	100 (5/5)	N/A	1.3±0.0 (5)
	10^8^	0 (0/15)* D	N/A	N/A	3.7±0.3 (15)	87 (13/15)	N/A	1.5±0.6 (15)
129S1/SvImJ	10^7^	0 (0/9)^F^	N/A	N/A	1.8±0.8 (9)	100 (9/9)	N/A	1.3±0.0 (9)
	10^8^	0 (0/10)* D	N/A	N/A	1.8±0.4 (10)	100 (10/10)	N/A	1.3±0.0 (10)

A Persistent Bacteriuria is defined as an individual mouse having greater than 10^4^ cfu UTI89 Kan^R^ per ml of free catch urine at every time point through 4 weeks of infection.

B Resolved Bacteriuria is defined as an individual mouse having less than 10^4^ cfu UTI89 Kan^R^ per ml of free catch urine at least once over 4 weeks of infection.

C Here PPV is the positive predictive value of persistent bacteriuria in predicting the occurrence of bladder titers >10^4^ cfu at 4 wpi, NPV is the negative predictive value of resolved bacteriuria in predicting the occurrence of bladder titers >10^4^ cfu at 4 wpi.

D 5 mice were infected with wild type UTI89 in initial studies and these data are included in the analysis.

E 25 mice infected, 5 mice died by 7 dpi.

F 4 mice were infected with wild type UTI89 in initial studies and these data are included in the analysis.

G 6 C3H/HeSnJ mice were infected with 10^8^ cfu wild type UTI89 in initial studies and these data are included in the analysis.

*Incidence of persistent bacteriuria is significantly different from C3H/HeN mice (*P*<0.05, two-tailed Mann-Whitney).

**†:** Titers significantly different from those from mice that resolved bacteriuria (*P*<0.05, two-tailed Mann-Whitney).

Limits of detection are Log_10_ transformed titers of 1.3-1.6 for bladders and 1.3 for kidneys.

Of the C57BL/6J and C3H/HeN mice that resolved bacteriuria, i.e. their urine titers fell to less than 10^4^ cfu UTI89 per ml at least once during the course of infection, 80 of 81 had bladder bacterial titers <10^4^ cfu at 4 wpi (Table 1; Fig. 1C–D). The presence of low level bladder bacterial burdens after acute infection, with urine and kidney titers at or near the limit of detection, is consistent with a quiescent intracellular reservoir (QIR) population comprised of bacteria in a dormant state [48], [49]. In contrast, C3H/HeN mice with persistent bacteriuria had significantly higher bladder and kidney titers at 4 wpi (Table 1; Fig. 1D). Furthermore, in these mice whole bladder titers exceeded kidney titers on average (geometric mean) by 2.9 orders of magnitude at the 10^7^ cfu inoculum, and increasing the inoculum did not significantly alter this bias towards bladder infection (*p* = 0.38, Mann-Whitney test). As a result, the development of persistent bacteriuria had a high positive predictive value (PPV) for the presence of high titer bladder infection (>10^4^ cfu) at 4 wpi (Table 1). In contrast, persistent bacteriuria in C57BL/6J mice was rare and when it did occur, severe kidney infection with abscess formation was a consistent finding in those mice (Table 1, data not shown).

Gross examination of the urinary tract organs of C3H/HeN mice at 4 wpi infection with either 10^7^ or 10^8^ cfu UTI89 (n = 58) revealed that all bladders from mice with persistent bacteriuria (n = 18) were enlarged and rigid compared to the bladders from mice that resolved bacteriuria (n = 40). Gross examination of the kidneys revealed no differences between disease outcomes, and kidney abscesses were not observed. Histopathological analysis of bladder tissue was performed on a random subset of mice (n = 17). Bladders from mice that had resolved bacteriuria (n = 9) lacked any evidence of cystitis (inflammation of the bladder) or noticeable pathology and the bladder epithelium appeared to be fully repaired (Fig. 1E). In contrast, abundant bacterial colonization accompanied by lesions of both acute and chronic inflammation, as defined by the presence of polymorphonuclear leukocytes (PMN, also called granulocytes) and mononuclear leukocytes, respectively, was detected in 7 of 8 bladders examined from mice with persistent bacteriuria (Fig. 1F). Acute inflammation was most apparent within the urothelium, with epithelial reactivity and marked infiltration by PMN that resemble neutrophils and other CD45^+^ cells. The bladder urothelium of persistently bacteriuric mice was hyperplastic and poorly differentiated, lacking the superficial facet cell layer as indicated by diminished uroplakin III expression on the luminal surface (Fig. 1G–H). Unlike the acute phase when UPEC progress through IBCs, bacterial colonization in these bladders appeared to be entirely luminal in nature as IBCs or other intracellular bacteria were not observed within the urothelium by immunofluorescence staining. Chronic inflammation was observed in the lamina propria (connective tissue layer underlying urothelium) with accumulations of lymphoid-like CD45^+^ cells in large follicle-like aggregates (Fig. 1F, I–J). These lymphoid follicles were found in all mice with persistent bacteriuria (n = 8 mice, 2–5 follicles/tissue section), while none were observed in the bladders of mice that resolved bacteriuria.

Similar pathology of bacterial colonization coupled with both acute and chronic inflammation were observed in the upper urinary tract of mice with persistent bacteriuria (data not shown, n = 4). However, these lesions were confined to tissues lined by urothelium: the ureters and renal pelvices, and were minimal or absent in the renal medullae and cortices. Thus, persistent bacteriuria in C3H/HeN mice through 4 wpi is highly indicative of chronic infection and inflammation of all urothelium-lined tissues and will be used in the following analyses as a method for identifying C3H/HeN mice with chronic cystitis.

### C3H/HeOuJ and C3H/HeJ mice differ in their susceptibility to chronic cystitis

**Figure 2:**
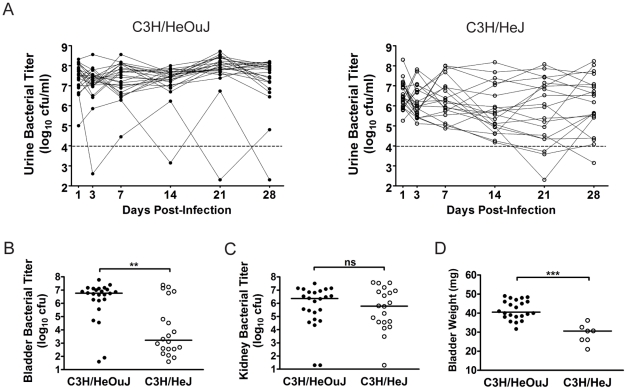
The outcome of UPEC infection of the urinary bladder differs between C3H/HeOuJ and C3H/HeJ mice. C3H/HeOuJ (closed circles) or C3H/HeJ (open circles) mice were infected with 10^7^ cfu UTI89 Kan^R^ and followed for 4 wpi. Data were compiled from two independent experiments. *A*, Urine bacterial titers were assayed over 4 wpi. Solid lines connect the urine titers over time for each individual mouse. Dashed horizontal line represents the cutoff for significant bacteriuria in free catch urines: 10^4^ cfu/ml. *B–C*, *B*, Bladder and *C*, Kidney titers were assayed at 4 wpi; *D*, Bladders with high bacterial burdens (>10^4^ cfu from panel *B*) were weighed at 4 wpi. All statistics in panels *B, C*, and *D* are the results of a two-tailed Mann-Whitney U test: **, *P*<0.01, ***, *P*<0.001 and **ns**, not significant; horizontal bars indicate median values.

The outcome of UPEC infection in C3H/HeOuJ and C3H/HeJ mice was also investigated, as these two C3H substrains have been reported to be highly susceptible to chronic bladder and kidney infection at 2 wpi with UPEC [45], [46]. We found that both C3H/HeOuJ and C3H/HeJ mice develop persistent bacteriuria at a much more efficient rate than C3H/HeN mice upon infection with 10^7^ cfu UTI89 Kan^R^ (Table 1, Fig. 2A). However, unexpectedly we found that C3H/HeJ mice, which are incompetent for TLR4 signaling, had significantly lower levels of bladder infection compared to C3H/HeOuJ mice at 4 wpi (Fig. 2B; Table 1). Yet, these strains were equally and highly susceptible to pyelonephritis compared to C3H/HeN mice (Fig. 2C, Table 1), often with grossly visible kidney abscess formation. Thus, persistent bacteriuria is a poor predictor of high bladder bacterial burdens (>10^4^ cfu) at 4 wpi in C3H/HeJ mice compared to either C3H/HeN or C3H/HeOuJ mice at 10^7^ cfu inoculum (PPV of 44% versus 100%, *P*<0.05, Fisher's Exact Test; Table 1). Finally, 5/25 C3H/HeJ mice died between 1 and 7 days post-infection (dpi), demonstrating the contribution of TLR4 signaling in preventing fatal bacterial infection.

**Figure 3:**
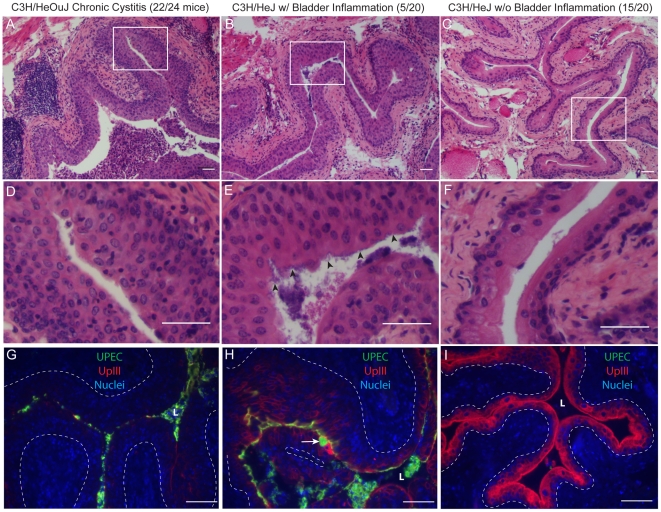
Chronic bladder infection in C3H/HeJ mice is histologically distinct from chronic cystitis in C3H/HeOuJ mice. Representative paraffin-embedded, fixed bladder sections from C3H/HeOuJ mice with chronic cystitis (22 out of 24 mice; panels *A, D, & G*), C3H/HeJ mice with high bacterial burdens and evidence of bladder inflammation (5 out of 20 mice; panels *B, E, & H*), and the remaining C3H/HeJ mice that uniformly lacked evidence of bladder inflammation (15 out of 20 mice; panels *C, F, & I*) were analyzed at 4 wpi; *Bars* approximate 50 µm in length. *A–F*, Sections were stained with hematoxylin & eosin and examined by light microscopy; *boxes* in panels *A–C* demarcate boundaries of higher magnification images in panels *D–F*. *G–I*, Sections were analyzed by indirect immunofluorescence (IF) microscopy, staining for *E. coli* (green) and uroplakin III (red), with nuclei counterstained with bis-benzimide (blue). “L” denotes bladder lumen; *dashed line* indicates approximate location of basement membrane; *arrowheads* point to urothelial surface bacterial colonization in panel *E*; and the *white* a*rrow* points to a mature superficial facet cell containing a possible IBC in panel *H*.

Among C3H/HeOuJ and C3H/HeJ mice with persistent bacteriuria and high bladder bacterial burdens (>10^4^ cfu) at 4 wpi, a number of differences were observed. The bladders from C3H/HeJ mice meeting these criteria (n = 7) weighed significantly less than those from C3H/HeOuJ mice (n = 22) (Fig. 2D). The bladders from C3H/HeOuJ mice with persistent bacteriuria demonstrated lesions of acute and chronic bladder inflammation (Fig. 3, panels A, and D) identical to those described above for C3H/HeN mice with persistent bacteriuria (see Fig. 1). These included marked luminal bacterial colonization and urothelial hyperplasia accompanied by extensive acute and chronic inflammatory cell infiltrates, highlighted by marked PMN infiltration and numerous lymphoid follicles. In contrast, histological evidence of bladder inflammation was only present in the 5 C3H/HeJ mice with the highest bladder bacterial burdens (>10^6^ cfu). Moreover, this inflammation, when present, was greatly subdued (Fig. 3, panels B, and E) compared to that observed in C3H/HeOuJ and C3H/HeN mice with high bladder bacterial burdens. Specifically, neutrophil infiltration from the mucosal tissue was reduced and lymphoid follicles were absent. For comparison, C3H/HeOuJ mice that resolved bacteriuria and C3H/HeJ mice with bladder colonization below 10^6^ cfu all lacked detectable bladder inflammation (Fig. 3, panels C and F, data not shown).

The urothelial response to high titer chronic UPEC infection of the bladder also differed between C3H/HeOuJ and C3H/HeJ mice. As with C3H/HeN mice, the terminal differentiated facet cell layer was entirely ablated in the C3H/HeOuJ mice with persistent bacteriuria and high titer bladder infections as indicated by the lack of uroplakin staining (Fig. 3G). In contrast, in the C3H/HeJ mice with persistent bacteriuria, high bladder titers, and evidence of bladder inflammation, the bladder mucosae retained a capacity for terminal differentiation, albeit reduced, as evidenced by the intermediate level of uroplakin expression compared to C3H/HeJ mice that lacked bladder inflammation (Fig. 3H and I). Furthermore, fully mature superficial facet cells with abundant uroplakin expression were detected in every bladder section, and a number of these harbored dense bacterial colonies surrounded by uroplakin staining, i.e. apparent IBCs (Fig. 3H, arrow). Neither mature superficial facet cells, nor IBCs were ever observed in the chronic stages of UTI in C3H/HeN or C3H/HeOuJ mice although IBCs were observed normally in the acute stages. We also observed that luminal colonization of the urothelium appeared much more robust in these 5 C3H/HeJ mice than in the other two C3H substrains, resembling biofilms (Fig. 3, panels E & H).

Therefore, in contrast to previous studies [45], [50], our data indicate that C3H/HeJ mice have significantly lower bladder bacterial burdens during chronic infection than C3H/HeOuJ mice, despite similar bacterial burdens in the kidneys. Furthermore, when bacterial persistence does occur in the bladders of C3H/HeJ mice, it may be mechanistically distinct from chronic cystitis in TLR4 signaling-competent C3H mice. Given that C3H/HeJ mice are known to be immunodeficient, mounting only very muted acute inflammatory responses to UPEC infection [30], [53], these results suggest that the host immune response may contribute to the development of chronic cystitis.

### Chronic cystitis is a common response in multiple, but not all, murine backgrounds to UPEC and *Klebsiella pneumoniae* infection of the urinary tract

We then investigated whether chronic cystitis was specific to UPEC infection in C3H/HeN and C3H/HeOuJ mice. We found that other C3H sub-strains, C3H/HeSnJ and C3HeB/FeJ, were also capable of developing chronic cystitis in response to infection with UTI89 (Table 1, Fig. S1A–B). However, the relative susceptibilities of these sub-strains to chronic cystitis differed: C3H/HeN mice were among the most resistant (18% at 10^7^ cfu) and C3H/HeOuJ mice were the most susceptible (92% at 10^7^ cfu). CBA/J mice, which are commonly used in experimental UTI models [54] and like C3H mice are descendents of a DBA by Bagg albino cross, and DBA/2J mice were each also susceptible to chronic cystitis (Table 1, Fig. S1C–D). In contrast, other common strains such as BALB/cJ and 129S1/SvImJ were not observed to develop chronic cystitis at either 10^7^ or 10^8^ cfu inoculum of UTI89 in this model (Table 1, Fig. S2), despite successful acute infections. Conversely, infection of C3H/HeN mice with either J96, another UPEC isolate, or Top52, a *Klebsiella pneumoniae* human cystitis isolate that had previously been demonstrated to form IBCs during acute infection [29], resulted in varying incidences of chronic cystitis (Fig. S3). Therefore, chronic cystitis in response to UPEC or *Klebsiella pneumoniae* infection appears to be restricted to certain strain backgrounds.

### Chronic cystitis can be predicted early in acute infection by serum cytokine biomarkers that correlate with the presence of urothelial injury

**Figure 4:**
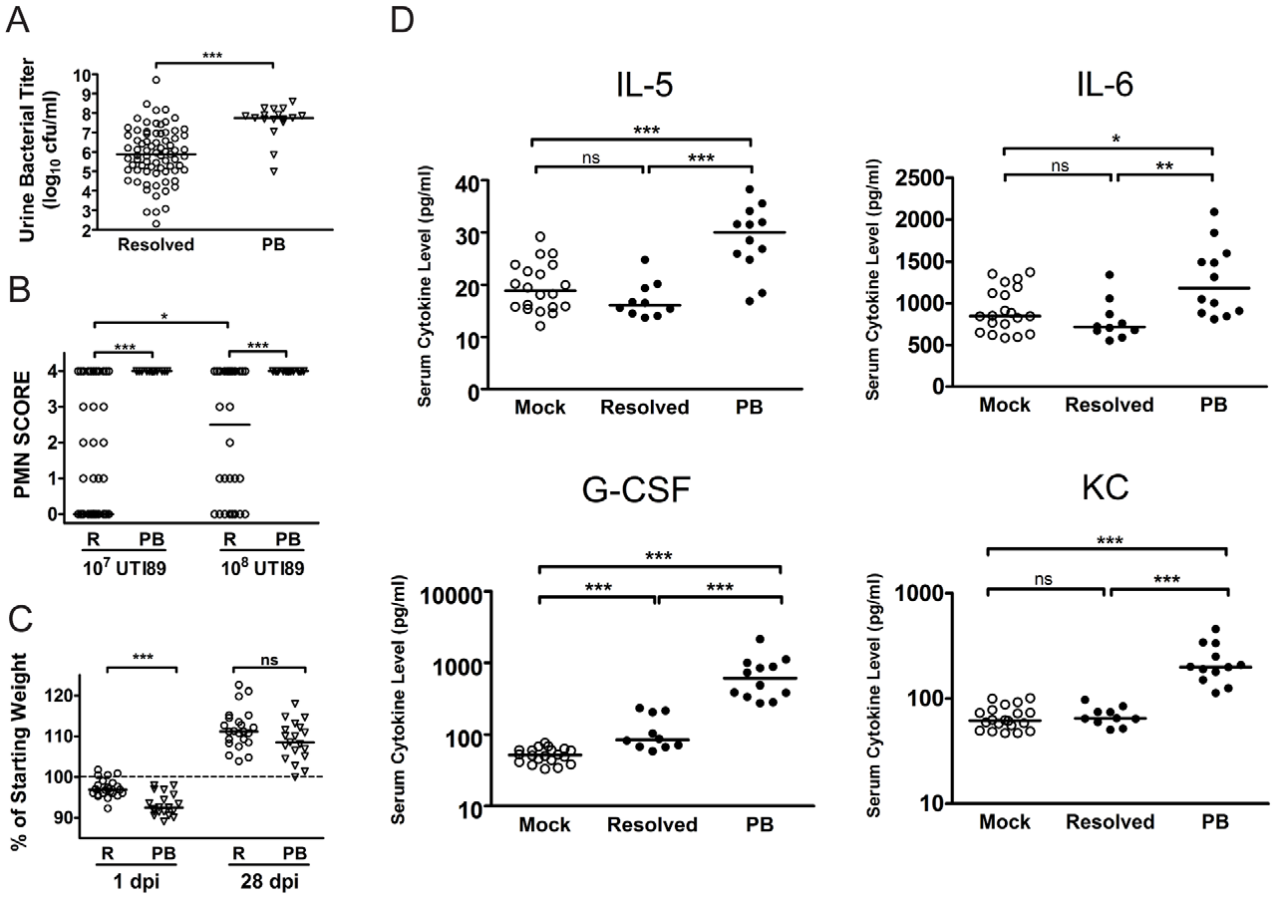
C3H/HeN mice that develop chronic cystitis can be distinguished from their cage mates by several parameters of infection and host response at 24 hpi. *A–C*, Parameters of acute infection and inflammation were assayed in mice that either resolved bacteriuria (**R**, open circles) or developed persistent bacteriuria (**PB**, open triangles). *A*, Urine bacterial titers were assayed at 24 hpi with 10^7^ cfu of UTI89 Kan^R^ or UTI89; data is compiled from seven independent experiments (n = 92 mice). *B*, Urine sediments at 24 hpi with either 10^7^ or 10^8^ cfu of UTI89 Kan^R^ or UTI89 were analyzed and assigned a semi-quantitative polymorphonuclear cell (PMN) score from 0–4 as explained in the Materials and Methods; data is compiled from 6 independent experiments (n = 123 mice). *C*, Mice were weighed at every urine collection time point in most studies. Plot depicts the weight loss or gain at 1 and 28 days post-infection (dpi) with 10^8^ cfu UTI89 Kan^R^; data is compiled from two independent experiments (n = 42 mice). *D*, Mouse sera collected at 24 hpi with either PBS **(Mock)** or 10^8^ cfu UTI89 Kan^R^ infected mice were analyzed for the presence of 23 cytokines by cytometric bead array as described in the Materials and Methods. UPEC infected mice were then grouped by disease outcome: resolved bacteriuria **(Resolved)**, or persistently bacteriuric **(PB)**. These four cytokines were significantly elevated in PB mice in each of three independent experiments; data shown is from one representative experiment. All statistics are by Mann-Whitney U two-tailed test: *, *P*<0.05, **, *P*<0.01, ***, *P*<0.001 and **ns**, not significant; horizontal bars indicate median values.

The relative resistance of TLR4 signaling-deficient C3H/HeJ mice, which have muted acute inflammatory responses [45], [50], [55], to chronic cystitis when compared to C3H/HeOuJ mice, which have very robust acute inflammatory responses to UPEC infection [45], [50], suggested a positive association between acute inflammation and chronic cystitis. To investigate this further, we tested whether the severity of the acute bladder inflammatory responses correlated with disease outcome in C3H/HeN mice. We found that mice that proceeded to develop persistent bacteriuria had significantly higher urine bacterial titers at 24 hours post-infection (hpi) than their cage mates that resolved bacteriuria (Fig. 4A). These mice also had increased numbers of PMN in the urine (pyuria), and greater weight loss at 24 hours post-infection (hpi) than their cage mates that resolved bacteriuria (Fig. 4B–C). However, this weight loss was transient and the mice quickly recovered to gain weight due to aging, indicating that chronic infection is well tolerated through 4 wpi. Severe acute pyuria and weight loss at 24 hpi was also predictive of chronic cystitis in C3H/HeOuJ mice, but was not observed in C3H/HeJ mice (Fig. S4A–B). To identify the nature of this acute systemic inflammatory response, we monitored the serum of mice at 24 hpi by multiplex cytometric bead array for the presence of 23 mouse cytokines. We found that C3H/HeN mice that developed persistent bacteriuria had significantly elevated levels of four serum cytokines at 24 hpi compared to both mock-infected mice and mice that resolved active infection: IL-5, IL-6, granulocyte colony-stimulating factor (G-CSF, encoded by Csf3), and keratinocyte-derived cytokine (KC, a.k.a. growth-regulated alpha protein, encoded by Cxcl1), an IL-8 analog in mice (Fig. 4D). Elevated levels of these serum cytokines at 24 hpi were also prognostic of chronic cystitis in C3H/HeOuJ, but not C3H/HeJ, mice (Fig. S4C). Another UPEC isolate, J96, also elicited similar serum responses prognostic of chronic cystitis in C3H/HeN mice (Fig. S5). Finally, we found that elevated urine IL-6, G-CSF, and KC, but not IL-5, were also predictive of the development of chronic cystitis in C3H/HeN mice at 24 hpi (Fig. S6)

**Figure 5:**
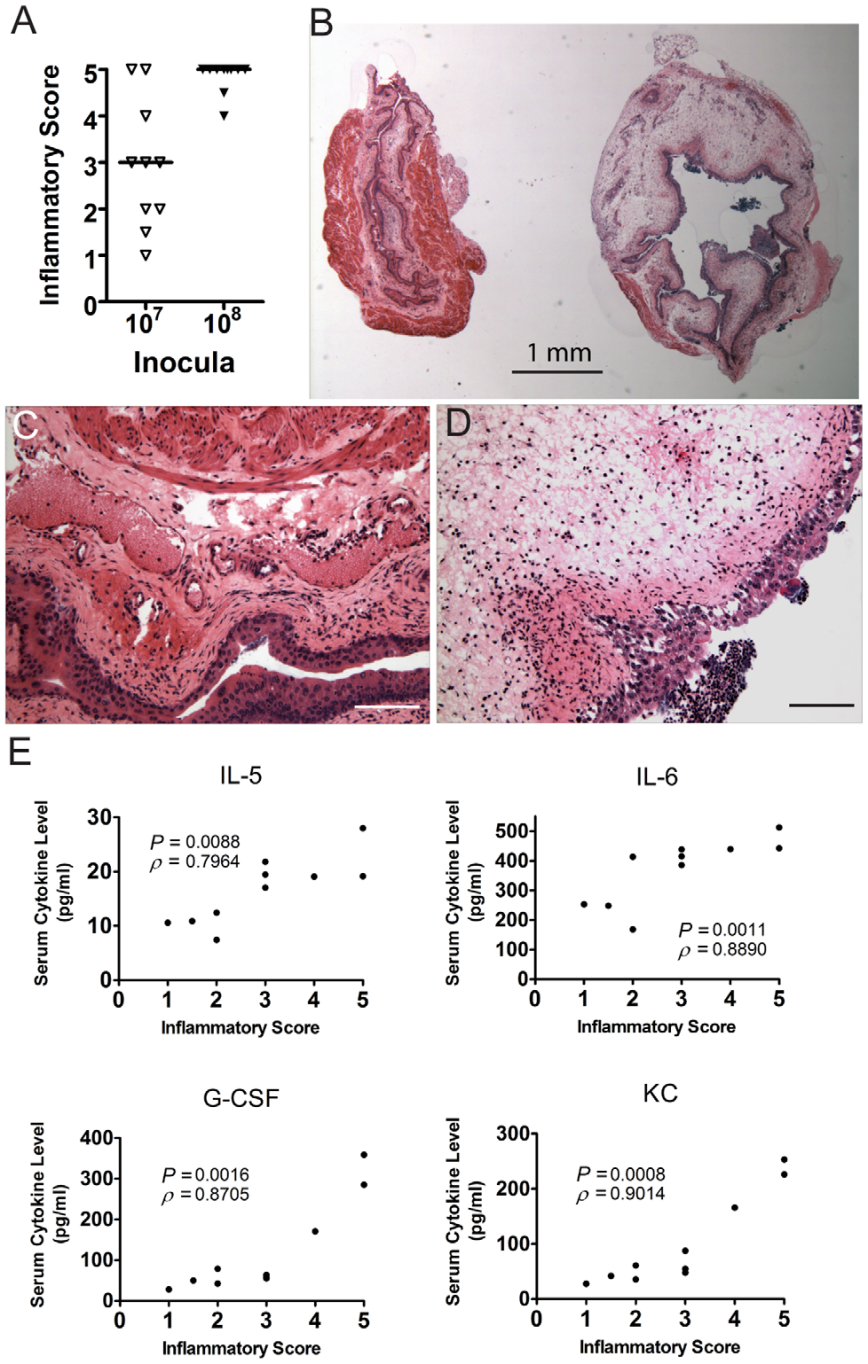
C3H/HeN mice that develop chronic cystitis have severe bladder inflammation at 24 hpi. Individual C3H/HeN mice were infected with either 10^7^ or 10^8^ cfu of UTI89 and bladders and sera collected at 24 hpi in two independent experiments. Data shown are from one representative experiment. *A*, Paraffin-embedded, fixed bladder sections were stained with hematoxylin & eosin and examined by light microscopy. Sections were scored from 0–5 to quantify the severity of inflammatory lesions as explained in the Materials and Methods; horizontal bars indicate median values. *B*, Examples are shown of the disparate response to UPEC infection in two individual mice from the same cage, infected on the same day with the same inoculum (10^7^ cfu UTI89). *C–D*, Higher magnification images of the two bladders shown in panel *B*. Inflammatory scores were 3 and 5 for panels *C* and *D*, respectively. *Bars* approximate 50 µm in length. *E*, Scatter plot analysis of serum IL-5, IL-6, G-CSF, and KC values, as labeled, versus the inflammatory scores of the bladders from the same individual mice. Significance (*P*) and strength (*ρ*) of correlations shown were determined by Spearman's rank correlation test.

At 24 hpi, the bladder inflammatory responses to acute UPEC infection vary widely among individual C3H/HeN mice infected with 10^7^ cfu UTI89 (Fig. 5A–D), despite the fact that the bacterial titers recovered from these bladders are fairly uniform at 6 hpi [24], [29]. Thus, we investigated the potential correlation between the severity of bladder inflammation and serum cytokine levels as prognosticators of chronic cystitis. C3H/HeN mice were infected with 10^7^ cfu UTI89. At 24 hpi we found that 20% of the mice had severely inflamed urinary bladders with full-thickness urothelial necrosis (inflammatory scores of 5), marked edema, epithelial necrosis, and inflammatory cell infiltrates (Fig. 5A, 5B (right) and 5D), while the other 80% of the bladders had minimal to moderate inflammation at 24 hpi (Fig. 5A, inflammatory scores of 1–4; 5B (left) and 5C). The incidence of severe inflammation was infectious dose-dependent, as 80% of mice had bladder inflammatory scores of 5 when infected with 10^8^ cfu (Fig. 5A). The levels of all four serum cytokines prognostic of chronic cystitis strongly and positively correlated with the degree of bladder inflammation at 24 hpi with 10^7^ cfu (Fig. 5E). Collectively, these findings demonstrate a strong association between severe, acute immunopathology and the development of chronic cystitis.

### C3H background severe combined immunodeficient mice are resistant to developing chronic cystitis

**Figure 6:**
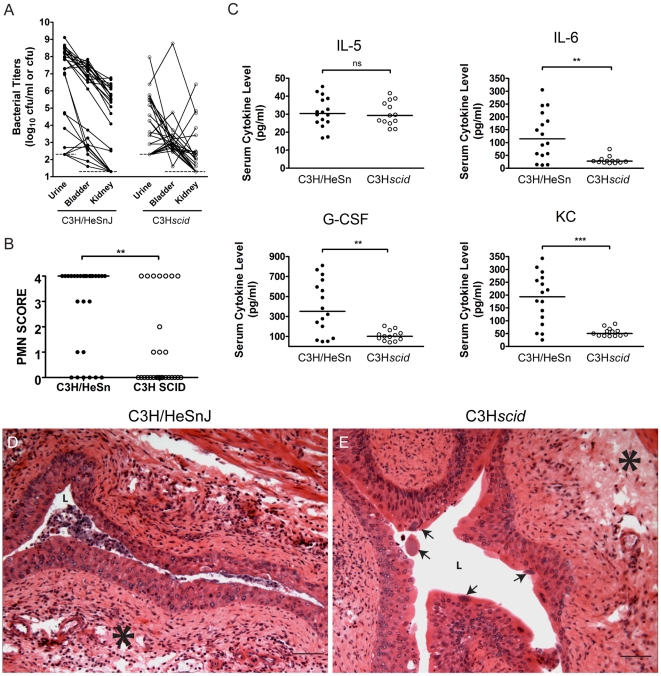
C3H background severe combined immunodeficient mice have muted acute inflammatory responses to UPEC infection and are resistant to chronic cystitis. C3H/HeSnJ (closed circles) or C3SnSmn.CB17-*Prkcscid*/J (C3H*scid*, open circles) mice were infected with 10^8^ cfu of UTI89 Kan^R^ or UTI89. *A*, Urine and tissue titers were assayed at 4 wpi; solid lines connect the different urine and tissue titers from the same mouse. *B*, PMN scores of the urine sediments were determined at 24 hpi. The data shown in panels A and B were compiled from four independent experiments. *C*, Sera were collected for cytokine analysis at 24 hpi. Data are representative of two independent experiments. All statistics in panels B–C are the results of the Mann-Whitney U test: **, *P*<0.01, ***, *P*<0.001 and **ns**, not significant, horizontal bars indicate median values. *D–E*, Representative paraffin-embedded, fixed bladder sections from *D*, C3H/HeSnJ, and *E*, C3H*scid* mice, at 24 hpi were stained with hematoxylin & eosin and examined by light microscopy. *Asterisks* indicate areas of submucosal edema, *arrows* indicate apparent IBCs, and *bars* approximate 50 µm in length.

To further substantiate whether the immune response is required for the development of chronic cystitis in C3H mice, we investigated the outcome of UPEC infection in C3H background severe combined immunodeficient (C3H*scid*) mice, which lack mature lymphocytes and may have other immune cell abnormalities. We found that C3H*scid* mice are almost entirely resistant to the development of chronic cystitis compared to the congenic wild type strain, C3H/HeSnJ (Figs. 6A, S7A). Even after infection with 10^8^ cfu of UTI89, only 4% of C3H*scid* mice developed persistent bacteriuria and high titer (>10^4^ cfu) bladder infection at 4 wpi, compared to 62% of C3H/HeSnJ mice (1/23 versus 16/26, *P*<0.0001, Fisher's Exact test). However, intermittent bacteriuria was common in the C3H*scid* mice, perhaps indicating a failure to eliminate colonization of specific niches. Nevertheless, intermittent bacteriuria was not associated with bladder bacterial burdens >10^4^ cfu at 4 wpi.

Acute inflammation was greatly muted in the C3H*scid* mice compared to their congenic wild type controls. PMN scores and weight loss at 24 hpi were significantly reduced (*P*<0.01 for each, Mann-Whitney test; Figs. 6B, S7B). Furthermore, while the C3H*scid* mice had a serum IL-5 response similar to wild type controls, they were completely unable to mount serum IL-6, G-CSF, and KC responses (Fig. 6C). Examination of the bladder tissue from 5 C3H*scid* and 5 wild type control mice at 24 hpi (Fig. 6D–E) revealed that these strains had similar levels of bladder edema (Fig. 6E). However, C3H*scid* mice entirely lacked the severe cellular inflammatory infiltrates and urothelial exfoliation seen in wild type C3H mice at 24 hpi, despite vigorous acute infection, including numerous apparent IBCs (Fig. 6E, arrows). Thus, the muted inflammatory response does not appear to result from a defect in acute colonization. These data provide further evidence that inflammation is required for the development of chronic cystitis and suggest that lymphocytes may play a necessary role in this process.

### The development of chronic bacterial cystitis in C3H/HeN mice is dependent upon severe acute inflammatory responses

**Figure 7:**
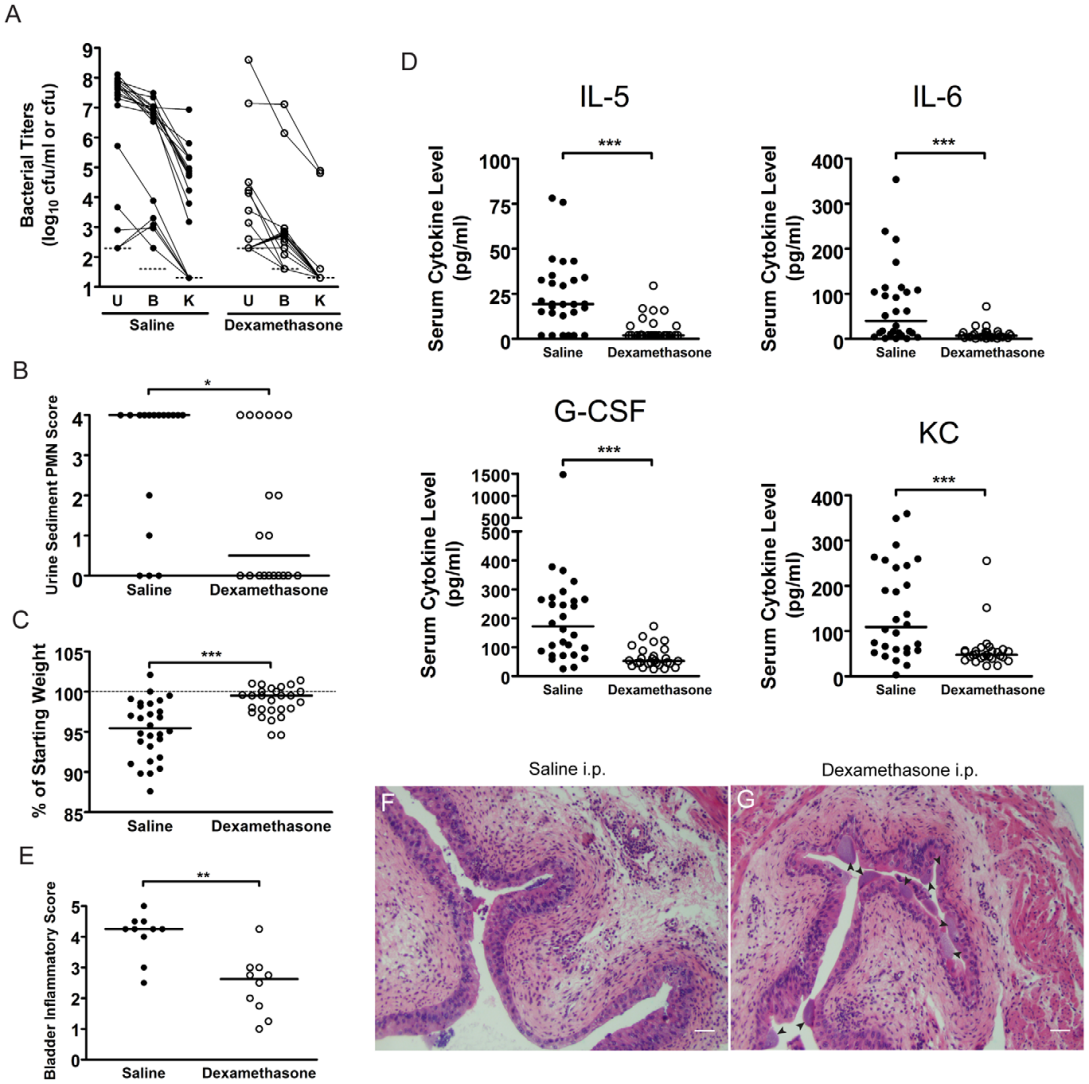
C3H mice treated with the glucocorticoid anti-inflammatory drug, dexamethasone, just prior to infection have muted acute inflammatory responses to UPEC infection and are protected against chronic cystitis. C3H/HeN mice were treated with either **saline** (closed circles) or 200 µg **dexamethasone** sodium phosphate (open circles) two hours prior to infection with 10^8^ cfu UTI89 Kan^R^. *A*, urine (**U**), bladder (**B**) and kidney (**K**) titers were determined at 4 wpi; solid lines connect the different urine and tissue titers from the same mouse. *B*, PMN scores of the urine sediments were determined 24 hpi. *A–B*, Data are compiled from three independent experiments. *C*, Percent weight change at 24 hpi was determined. *D*, Serum cytokine analysis of mice at 24 hpi with 10^8^ cfu of UTI89 Kan^R^ is depicted. *C–D*, Data are compiled from four independent experiments. *E–G*, C3H/HeN mice pre-treated with either saline or dexamethasone were sacrificed at 24 hpi and paraffin-embedded, fixed bladder sections were stained with hematoxylin & eosin and examined by light microscopy. Semi-quantative bladder inflammatory scores are plotted in panel *E*, and representative images of bladders from mice treated with *F*, saline, and *G*, dexamethasone, are depicted; *arrowheads* indicate apparent IBCs; *bars* approximate 50 µm in length. All statistics in panels *B–E* are the results of the Mann-Whitney U test: *, *P*<0.05, **, *P*<0.01, and ***, *P*<0.001; horizontal bars indicate median values.

To test whether severe acute inflammation is specifically required for the development of chronic cystitis, we treated C3H/HeN mice with a single immunosuppressive dose of the glucocorticoid, dexamethasone sodium phosphate, by intraperitoneal injection 2 hours prior to infection. We found that mice treated with dexamethasone were significantly more resistant to the development of chronic cystitis (Figs. 7A, S8A) upon infection with 10^8^ cfu UTI89 Kan^R^ compared to saline-treated controls (2/17 versus 13/18, *p*<0.001, Fisher's Exact test). Furthermore, dexamethasone treatment significantly reduced the severity of acute inflammation at 24 hpi in reponse to UPEC infection as indicated by pyuria (Fig. 7B), weight loss (Fig. 7C), serum cytokines (Fig. 7D), and bladder inflammation (Fig. 7E–G). Yet, these groups had similar bladder bacterial burdens at 24 hpi (Fig. S8B), including what appears to be robust IBC formation in the dexamethasone treated group (Fig. 7G, arrows). Thus, the development of chronic cystitis appears to require severe acute inflammation, strongly suggesting that bladder immunopathology during acute infection facilitates the establishment of chronic infection.

### The development of chronic bacterial cystitis in juvenile C3H/HeN mice predisposes them to chronic infection upon subsequent challenge with UPEC

**Figure 8:**
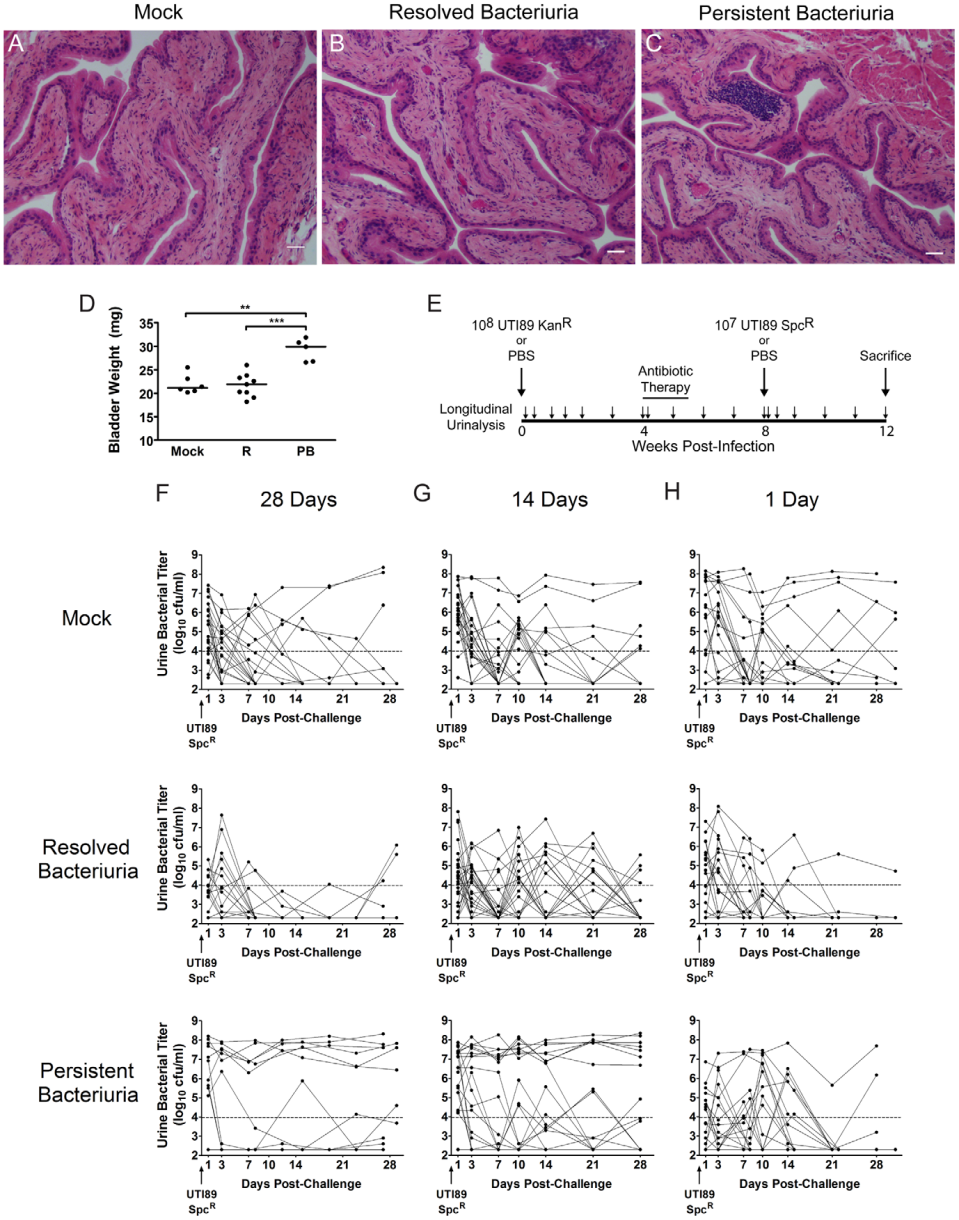
C3H/HeN mice with a history of chronic cystitis are more susceptible to chronic cystitis upon challenge infection. *A–D*, Mice were infected with 10^7^ UTI89 Kan^R^ or mock-infected with PBS and followed for 4 wpi by longitudinal urinalysis in two independent experiments. All mice were treated with antibiotics at 4 wpi and sacrificed 4 weeks after initiation of antibiotic therapy. Paraffin-embedded, fixed bladder sections were stained with hematoxylin & eosin and examined by light microscopy. Representative images of mice that *A*, were mock-infected, *B*, had resolved bacteriuria, or *C*, had persistent bacteriuria are shown; *bars* approximate 50 µm in length. Statistics are the results of the Mann-Whitney U test: **, *P*<0.01, and ***, *P*<0.001. *D*, Bladders were weighed at sacrifice; horizontal bars indicate median values. *E*, Experimental time line for panel *F*: mice were followed by longitudinal urinalysis (each urine collection is indicated by small *arrow*) for the entire experiment to identify persistently bacteriuric mice prior to antibiotic therapy and after re-infection, and to confirm clearance of bacteriuria with antibiotic therapy. *F–H*, Combined time courses from two independent experiments each are displayed, charting the UTI89 Spc^R^ urine bacterial titers during the 4 weeks after challenge infection, when antibiotic therapy was initiated at *F*, 28 days, *G*, 14 days, or *G*, 1 day after the initial infection. In all cases, mice were challenged 4 weeks after the initiation of antibiotic therapy. For panels *F* and *G*, mice are grouped by the outcome of urinalysis during the initial infection: **Mock**, mock-infected with PBS at week 0; **Resolved Bacteriuria**, infected with UTI89 Kan^R^ at week 0 and subsequently had at least one time point with <10^4^ cfu/ml UTI89 Kan^R^; or **Persistent Bacteriuria**, infected with UTI89 Kan^R^ at week 0 and had >10^4^ cfu/ml UTI89 Kan^R^ for each time point. For panel *H*, mice are grouped by serum KC value at 24 hpi with UPEC: **Mock**, mock-infected with PBS at week 0; **Resolved Bacteriuria**, infected with UTI89 Kan^R^ at week 0 and have serum KC in the lower 50% of values at 24 hpi; or **Persistent Bacteriuria**, infected with UTI89 Kan^R^ at week 0 and had serum KC in the upper 50% of values at 24 hpi. Solid lines connect the urine titers over time for each individual mouse. Horizontal dashed lines represent the cutoff for significant bacteriuria in free catch urines: 10^4^ cfu/ml.

Lymphoid follicles have been reported in humans with persistent bacteriuria, with or without symptoms of UTI, and have been reported to resolve spontaneously after antibiotic therapy to clear infection [56], [57]. We also found that persistent bacteriuria in C3H/HeN mice was rapidly eliminated by antibiotic therapy initiated at 4 wpi (n = 46). Bladder tissue sections from a sample of these mice (n = 5), together with mock-infected controls (n = 6) and mice that had resolved bacteriuria (n = 9), were examined one month after initiation of antibiotic therapy. This analyisis revealed that urothelial integrity was indistinguishable between these groups, indicating full healing of the urothelium in the previously infected mice (Fig. 8A–C). However, the bladders of mice with a history of persistent bacteriuria weighed significantly more than either of the other two groups (Fig. 8D), and while the lymphoid follicles were largely gone, small to moderately sized clusters of mononuclear cells remained in the bladder lamina propria (Fig. 8C). Similar chronic inflammatory cell infiltrates have been reported after serial UPEC infections of C57BL/6J mice, where their presence coincides with the development of adaptive immunity [9].

These findings suggested that an acquired, bladder-resident immune cell population may modulate the host response to subsequent infection. Therefore, we tested whether the specific disease outcome, persistent bacteriuria or resolved bacteriuria, affected the susceptibility of mice to UTI challenge after clearance of infection with antibiotics, compared to age-matched naïve mice (Fig. 8D). All challenges were performed 4 weeks after initiation of antibiotic therapy, which allowed time for the bladder urothelium to heal fully as described above (Fig. 8A–C). We found that mice with a history of persistent bacteriuria for 4 wpi with UTI89 Kan^R^ at 7–8 weeks of age were significantly more prone to developing persistent bacteriuria (Fig. 8E) and chronic cystitis (Table 2) upon challenge infection with an isogenic, spectinomycin-resistant UTI89 strain, UTI89 Spc^R^, at 15–16 weeks of age than either their cage mates who spontaneously resolved bacteriuria or age-matched naïve mice that had previously been mock-infected. Redevelopment of chronic cystitis was significantly associated with the onset of severe pyuria and elevated serum IL-5, G-CSF, and KC at 24 hpi, compared to mice that resolved the challenge infection, whether or not they had a history of chronic cystitis (data not shown). The rapid clearance of bacteriuria after bacterial challenge in mice that had previously spontaneously resolved infection, compared to naïve mice, suggests that these mice develop some degree of acquired immunity to UPEC. Only 5% (2/42) of mice previously infected with UTI89 Kan^R^ had recurrent bacteriuria with this strain on the day of challenge, 4 weeks after initiation of antibiotic therapy, similar to what was previously reported in C57BL/6J mice [46]. Otherwise, little reemergence of UTI89 Kan^R^ was observed upon challenge with UTI89 Spc^R^ (data not shown). As additional controls, a subset of 20 mice, 10 previously infected and 10 age-matched naïve, and all treated with the antibiotic regimen described above, were challenged with PBS and their urines remained free of UPEC for the remainder of the experiment (data not shown).

**Table 2:** Incidence of chronic cystitis upon challenge infection.

Outcome of Initial Infection^A^	Duration of Initial Infection Prior to Antibiotic Therapy		
	28 days	14 days	1 day^A^
**Mock-Infected**	1/24	2/25	4/20
	4%	8%	20%
**Resolved Bacteriuria**	0/16	0/30	0/21*
	0%	0%	0%
**Persistent Bacteriuria**	6/15†	7/20††	1/21
	40%	35%	6%

A For mice infected only one day, individuals with serum KC values below the median value at 24 hpi were assigned to the Resolved Bacteriuria group and those with serum KC values above the median value at 24 hpi were assigned to the Persistent Bacteriuria group.

**†:** *p*<0.01 compared to both Mock-Infected and Resolved Bacteriuria groups, Fisher's Exact test.

**††:** *p*<0.001 compared to Resolved Bacteriuria group, Fisher's Exact test.

*p<0.05 compared to Mock-Infected group, Fisher's Exact test.

To determine the duration of infection sufficient for modulating the host susceptibility to further infection, we initiated antibiotic treatment at 24 hpi and 2 wpi and then challenged mice with UPEC four weeks later, as described above (Table 2, Fig. 8F–G). We found that 14 days of chronic infection were sufficient for inducing the hypersusceptibility phenotype to challenge infection, but 1 day of infection was not. This result indicates that, although accurate indicators of the development of chronic cystitis exist at 24 hpi, infection-related events in the course of sub-acute to chronic UTI are necessary for the enhanced susceptibility to further chronic infections. Furthermore, these findings refute the alternative explanation to our data: that we are selecting for a pre-existing subpopulation, either due to the presence of genetic polymorphisms within the breeding colony, or prior exposure to pathogens. Taken together, these data indicate that the development of chronic UPEC cystitis predisposes C3H mice to chronic UTI upon subsequent colonization.

## Discussion

Using a C3H murine model of UTI, we have unveiled an inflammation-dependent checkpoint occurring at the host-pathogen interface within the first 24 hours of infection that has significant ramifications upon the long term fate of disease. Furthermore, subsequent development of chronic cystitis alters the host susceptibility to further bacterial cystitis after antibiotic therapy, resulting in increased susceptibilities to both severe acute symptomatology and chronic infections. We identified elevated IL-5, IL-6, G-CSF, and KC as a serum biomarker signature prognostic of the development of chronic cystitis in C3H mice. This signature was accompanied by severe bladder inflammation at 24 hpi as indicated by marked bladder edema and pyuria. While the severity of bladder damage and systemic signs diminish after the early acute stage of infection, these mice remain persistently bacteriuric and develop chronic cystitis. This improvement in clinical signs despite persistent bacteriuria is similar to the natural course of UTI reported in a majority of women from placebo-controlled studies [51], [52]. The alternate host response was characterized by mild to moderate bladder inflammation in the acute stage with minimal or no systemic signs. These mice typically achieved resolution of acute bacteriuria within 1–2 weeks, though recurrent bacteriuria after this time was common, either because of transient periurethral colonization or rUTI. The fate of disease outcome appears to be determined within the first 24 hpi and is infectious dose-dependent, indicating that this response is at least partially stochastic. Understanding the molecular and cellular mechanisms of this acute checkpoint may have far-reaching implications for how UTI and other chronic and recurrent infections are treated and evaluated.

Our findings indicate that a glucocorticoid-sensitive, pro-inflammatory response to early acute infection is required for the efficient development of chronic cystitis. Glucocorticoids are made in the adrenal gland and, among other functions, act as potent anti-inflammatory agents. Specifically, they block the production of acute mediators of inflammation such as TNFα, IL-1, IL-6, and arachidonic acid derivatives such as prostaglandins and leukotrienes, in part through blockade of NF-κB activity [58]. For example, dexamethasone is commonly used as an adjunct therapy to treat acute bacterial meningitis, as its anti-inflammatory activity appears to improve survival [59]. The immunosuppressive effects of a single dexamethasone treatment are transient, providing evidence for a mucosal immune checkpoint early in UPEC infection that, when triggered, leads to urinary tract immunopathology and facilitates chronic cystitis.

The resistance of C3H*scid* mice to chronic cystitis implicates lymphocytes as immune cells necessary for the development of chronic cystitis in C3H mice. Specifically, our data suggest that one or more lymphocyte populations with innate properties may play a necessary role in mediating a number of the early severe inflammatory responses to UPEC that are associated with the development of chronic cystitis. Candidate lymphocytes include γδ T cells, natural killer T (NKT) cells, and B1 cells, which make natural antibodies. γδ T cells have previously been implicated in host resistance to UTI in C57BL/6J mouse infection models [34], [41], and they are normally found in the bladder lamina propria of naïve mice [60]. Acute host responses that were muted or absent in C3H*scid* mice included severe pyuria, weight loss, and elevation of serum IL-6, G-CSF, and KC. Induction of both IL-6, an acute phase pro-inflammatory cytokine that causes fever and malaise if systemic, and IL-8, a chemotactic cytokine for granulocytes, are strongly associated with UTI in both humans and mice [36], [37], [61], [62], [63]. The combination of elevated IL-6, KC, an IL-8 analog, and G-CSF, a growth factor that promotes granulocyte development and release from the bone marrow, in both the serum and urine would explain the weight loss and severe acute PMN response associated with the development of chronic cystitis in mice [64]. Yet, these acute molecular and cellular responses are inadequate for resolving infection in C3H mice. Furthermore, a recent study suggests that such a response may contribute to disease pathogenesis, as depletion of G-CSF surprisingly resulted in lower UPEC titers in the bladders of C57Bl/6J mice at 48 hpi, despite reduced neutrophil infiltration into the bladder [33]. Similarly, treatment with G-CSF exacerbates disease in a murine model of *Klebsiella pneumoniae* respiratory tract infection [65]. Further studies are needed to identify the immune cell populations required for the development of chronic cystitis, and to clarify the role of these early innate responses in disease pathogenesis.

We have found a strong association between elevated serum IL-5 at 24 hpi and the development of chronic cystitis. IL-5 promotes eosinophil and B1 lymphocyte development and IgA class switching and has been reported to enhance production of IL-6 in kidney cell lines [66], [67]. However, the role of IL-5 in UTI pathogenesis is unclear. While “low level” IL-5 mRNA expression has been reported in the mouse bladder early in UPEC infection of C3H/HeN mice, IL-5 has not otherwise been implicated in UTI pathogenesis [68]. T helper cells polarized to produce type 2 cytokines (T_H_2 cells) have long been considered the primary source of IL-5, but the acute serum IL-5 response to UPEC infection in this study was still present in C3H*scid* mice. This finding is consistent with a previous study in mice, which found that non-lymphoid cells, such as mast cells or basophils, are the major source of IL-5 in the peripheral organs [69]. Activation of either of these cell types could mediate the bladder edema seen in C3H*scid* mice. However, if IL-5 is originating from the bladder, it does not appear to be able to access the bladder lumen as IL-5 was not elevated in the urines of mice at 24 hpi, regardless of disease outcome.

C3H background mice are known to have differing susceptibilities to various bacterial infections [70], [71], [72], [73]. Currently, the specific genetic basis for susceptibility to chronic cystitis and chronic pyelonephritis are unknown. However, a recent mouse genetics study by Hopkins and colleagues has made significant progress in identifying genetic loci associated with susceptibility to cystitis and pyelonephritis at 10 dpi in second generation crosses of C3H/HeJ (reported as susceptible to each) and Balb/c (resistant) mice [74]. In those studies, the authors utilized quantitative trait loci (QTL) analyses to discover that the genetic basis for chronic cystitis and pyelonephritis differed. This is consistent with our data comparing experimental UPEC infection in the various C3H sub-strains through 4 wpi, which demonstrate that chronic cystitis and chronic pyelonephritis can be independent disease outcomes. In C3H/HeN, C3H/HeSnJ, and C3HeB/FeJ mice with chronic cystitis, concurrent kidney infection was an inconsistent finding and, when present, was largely limited to the renal pelvis (pyelitis). Equally, the majority of C3H/HeJ mice with chronic pyelonephritis did not have cystitis at sacrifice, as defined by the presence of bladder inflammation and bacterial titers greater than 10^4^ cfu. Only in C3H/HeOuJ mice was there a strong association between chronic cystitis and pyelonephritis.

Since previous work had demonstrated that UPEC infection of both C3H/HeJ and C3H/HeOuJ mice resulted in similarly high bladder titers at 14 dpi, it had been hypothesized that chronic cystitis occurred independently of TLR4 signaling [45], [50]. However, our studies have now demonstrated that these two strains differ in their susceptibility to chronic cystitis. We suggest that differences in experimental methodology, including the use of ten-fold higher inocula and study durations limited to 2 weeks, likely prevented resolution of the differing susceptibilities of C3H/HeOuJ and C3H/HeJ mice to chronic cystitis in previous studies [45], [50]. In support of the hypothesis that enhanced TLR4 signaling contributes to the development of chronic cystitis, which in turn enhances host susceptibility to recurrent symptomatic infection, a TLR4 polymorphism that results in diminished responses to LPS has recently been associated with reduced susceptibility to rUTI in humans [75]. We also found that a subset of C3H/HeJ mice developed a persistent biofilm-like bladder colonization in the absence of robust inflammation. Bacterial biofilms and reduced TLR4 expression on neutrophils have each been associated with asymptomatic bacteriuria in humans [40], [76]. Thus, the innate host responses in the urinary tract must be fine-tuned to successfully eliminate UPEC infection while also maintaining tissue integrity.

Intracellular bacterial communities (IBC) had not been previously observed during the chronic stage of UTI and thus their role in bacterial persistence was unknown. However, IBC formation is known to be required during the acute stages of infection [17], [23], [24], [25], [26], [27], [28], [29] which is a prerequisite for subsequent chronic infection. For example, we recently identified residues in FimH, the mannose-binding tip adhesin subunit of type 1 pili, that are under positive selection in UPEC strains isolated from human patients with UTI and that function in IBC formation [26]. A double mutation in two of these residues abolished IBC formation despite retaining the ability to bind to mannose, bind to the urothelium, and invade the urothelium. This mutant was severely defective in a mouse model of UTI, and behaved similarly to a *fimH* knockout or a FimH receptor binding mutant. Therefore, this mutant separated the phenotypes of (i) mannose-inhibitable binding and invasion of the urothelium and (ii) IBC formation [26]. Its phenotype in a mouse model of UTI indicates that both of these phenotypes are critical for acute stages of UTI, which we have now shown are a prerequisite for persistence. However, the lack of development of chronic cystitis in C3H*scid* mice and in C3H mice treated with dexamethasone was not due to the lack of IBC formation in the acute stages of infection since IBCs developed normally in the acute stages of infection in these mice. Furthermore, we found evidence of IBC formation in the chronic stage of infection in C3H/HeJ mice, which have muted inflammatory responses to UPEC infection. This finding implicates mucosal inflammation, including urothelial reactivity and exfoliation, in restricting continuous IBC formation in immunocompetent mice. It also raises the hypothesis that one mechanism of asymptomatic bacteriuria, for which UPEC infection of C3H/HeJ mice has been proposed as a model [77], may be continuous IBC formation in hosts with muted urothelial inflammatory responses to UPEC.

In summary, we have discovered a new basis for understanding UTI that provides a possible mechanism for both chronic and recurrent infection. We propose that, in females that are genetically predisposed to enhanced mucosal TLR4 signaling, initial episodes of UTI due to UPEC or *Klebsiella pneumoniae* may be particularly severe in both degree of symptomatology and duration. If allowed to progress past the early acute stage before initiation of antibiotic therapy, these individuals could then develop altered bladder mucosal responses to gram-negative uropathogens. Upon repeated exposure to gram-negative uropathogens, these individuals would then be at increased risk for developing severe, symptomatic rUTI. It is also possible that a quiescent intracellular reservoir (QIR) state may exist in humans, similar to what has been described in mice [47], [49], not only serving as seeds for recurrent UTI, but also potentially modulating the mucosal response to UPEC due to the chronic persistence of bacteria within mucosal cells. This hypothetical model not only provides a logical basis for further, intensive murine and human UTI studies, but also extends our general understanding of chronic and recurrent gram-negative infections of mucosae.

## Materials and Methods

### Ethics statement

All animal experimentation was conducted following the National Institutes of Health guidelines for housing and care of laboratory animals and performed in accordance with institutional regulations after pertinent review and approval by the Animal Studies Committee at Washington University School of Medicine.

### Bacterial strains and cultivation

The UPEC strains used in this study were the human cystitis isolate, UTI89 [17] and derivatives thereof: UTI89 *att_HK022_*::*COM-GFP* (kanamycin-resistant, Kan^R^) and UTI89 *attλ*::*PSSH10-1* (spectinomycin-resistant, Spc^R^) [78]; and the human pyelonephritis isolate, J96 [79]. The *Klebsiella pneumoniae* strain used, TOP52, is a human cystitis isolate [29]. Bacteria were routinely cultured in Luria-Bertani (LB) broth.

### Mouse infections

C3H/HeN mice were obtained from Harlan Sprague Dawley, Inc. (Indianapolis, IN). 129S1/SvImJ, Balb/cJ, C3HeB/FeJ, C3H/HeJ, C3H/HeOuJ, C3H/HeSnJ, C3Smn.CB17-*Prkdc^scid^*/J, C57BL/6J, CBA/J and DBA/2J mice were all obtained from the Jackson Laboratory (Bar Harbor, ME). Bacterial strains were inoculated into 20 mL of LB broth directly from freezer stock, grown statically at 37°C overnight, and subcultured 1∶1000 into 20 ml of fresh media and again grown statically at 37°C for 18 hr. These cultures were centrifuged for 10 min at 3000×g, resuspended in 10 ml PBS, and then diluted to approximately 2–4×10^8^colony forming units (cfu)/ml (OD_600_ = 0.35). 50µL of this suspension (∼1–2×10^7^ cfu) or one concentrated 10-fold (∼1–2×10^8^ cfu) was inoculated into the bladders of 7–8 week old female mice by transurethral catheterization.

### Urine collection, bacterial titering, and urine sediment analysis

In most cases urines were collected prior to infection, at 1, 3, 7, 10, and 14 dpi, and then weekly thereafter by applying suprapubic pressure with proper restraint and collecting the urine stream in sterile 1.5ml eppendorf tubes. Urines were then serially diluted in PBS and 50 µL total of each dilution was spotted onto LB, and LB with 25 µg/ml kanamycin (LB/Kan25) where appropriate. In experiments where a marked strain was used, urine titers were always reported from the plate containing the relevant antibiotic. Urine sediments were obtained by cytocentrifuging 80 µL of a 1∶10 dilution of the collected urine onto poly-L-lysine-coated glass slides and stained as described [28]. Stained urine sediments were examined by light microscopy on an Olympus BX51 light microscope (Olympus America), and the average number of polymorphonuclear leukocytes (PMN) per 400× magnification field (hpf) calculated from counting 5 fields. A semi-quantitative scoring sytem was created to facilitate analysis: **0**, less than 1 PMN/hpf; **1**, 1–5 PMN/hpf; **2**, 6–10 PMN/hpf; **3**, 11–20 PMN/hpf, and **4**, >20 PMN/hpf.

### Tissue bacterial titer determinations

To quantify the bacteria present in urinary tract tissues at the time of sacrifice, bladders and kidneys were aseptically harvested at the indicated time point and homogenized in PBS. Homogenates were then serially diluted and spotted as described above, duplicate plating on LB and LB/Kan25 where appropriate. Tissue homogenates to be analyzed later for the presence of soluble cytokines were centrifuged at high speed for 5′ at 4°C and the supernatant removed for storage at −80°C.

### Serum collection and storage

Venous blood was collected by submandibular puncture using 5 mm steel lancets (Medipoint, Inc., Mineola, NY) into BD Microtainer serum separation 400 µl tubes. Blood tubes were allowed to clot at room temperature for 1–2 hours and, after centrifugation at 15,000×g for 5′, were stored at −20°C.

### Histopathology and immunofluorescence

Tissues were either fixed in methacarn (60% methanol, 30% chloroform, 10% glacial acetic acid) or embedded in OCT and frozen on dry ice before long term storage at −80°C. Methacarn fixed tissues were embedded in paraffin, sectioned, and stained with hemotoxylin and eosin. Bladder inflammatory scores were determined in a blinded fashion by two investigators (T.J.H. and C.S.H.) and an average score calculated, as previously described [45]. 7µm thick frozen sections were cut and fixed in acetone at −20°C for 10 minutes. All sections were hydrated and blocked in 1% BSA, 0.3% triton X-100 in PBS. After incubation with primary and secondary antibodies and associated washes, slides were stained with bis-benzimide (Sigma). Stained tissues were examined by epifluorescence microscopy on a ZEISS Axioskop 2 MOT Plus microscope.

### Serum and urine cytokine analysis

The presence of 23 mouse cytokines was analyzed in specimens by a Luminex-based multiplex cytometric bead array platform (Bioplex, Bio-Rad, Hercules, CA). These cytokines were IL-1α, IL-1β, IL-2, IL-3, IL-4, IL-5, IL-6, IL-9, IL-10, IL-12 (p40 subunit), IL-12 (p70 subunit), IL-13, IL-17, Eotaxin, G-CSF, granulocyte-macrophage colony-stimulating factor (GM-CSF), gamma interferon, KC, monocyte chemotactic protein 1 (MCP-1), macrophage inflammatory protein (MIP) 1α, MIP-1β, Regulated upon Activation, Normal T-cell Expressed and Secreted (RANTES), and tumor necrosis factor α. Individual samples were run in duplicate and the mean values used in all graphs.

### Glucocorticoid treatment

Dexamethasone sodium phosphate (Dexaject SP, Butler Animal Health Supply, Dublin, OH) was diluted to 1 mg/ml in sterile saline and one group of mice were given 200 uL of this solution intraperitoneally (i.p.), corresponding to a dose of 20 µg (10 mg/kg), two hours prior to UPEC infection, as previously described [80], [81]. Another group of control mice were mock-treated i.p. with sterile saline alone.

### UPEC challenge infections

At 1, 14, or 28 dpi with either PBS or 10^8^ cfu of Kan^R^, all mice were treated with trimethoprim and sulfamethoxazole in the drinking water daily for 10 days at concentrations of 54 and 270 µg/ml, respectively [46]. During this time, longitudinal urinalysis was continued weekly to confirm clearance of bacteriuria. Four weeks after the initiation of antibiotic therapy, mice from each test group (previously infected or naïve) were challenged with either PBS or 10^7^ cfu of UTI89 Spc^R^. Longitudinal urinalysis was then performed as for the primary infection (except now triplicate plating on LB, LB/Kan25 and McConkey agar with 50 µg/ml spectinomycin (McC/Spc50) to identify mice with persistent bacteriuria and the responsible strain. Mice were sacrificed 4 weeks after challenge and tissue titers determined as above, triplicate plating on LB, LB/Kan25, and LB/Spc50.

### Statistical analysis

Statistical analyses were performed using GraphPad Prism and InStat (GraphPad Software) and significance was defined by attaining *P* values<0.05, in two-tailed tests where appropriate.

### UniProt accession numbers for proteins mentioned in the text

Mouse Interleukin 5: P04401; Mouse Interleukin 6: P08505; Mouse Granulocyte Colony Stimulating Factor: P09920; Mouse Keratinocyte-derived Cytokine (Growth-regulated alpha protein): P12850.

## Supporting Information

Figure S1 Numerous C3H and related inbred mouse strains are susceptible to chronic cystitis with the UPEC strain, UTI89. Time course of bacteriuria and tissue titers were assayed after 4 weeks of infection with 10^7^ or 10^8^ cfu of either UTI89 Kan^R^ or UTI89 as indicated in Table 1, in *A*, C3H/HeSnJ, *B*, C3HeB/FeJ, *C*, CBA/J and *D*, DBA/2J mice. Solid lines connect the urine titers over time for each individual mouse. Dashed horizontal lines in time courses represent the cutoff for significant bacteriuria in free catch urines: 10^4^ cfu/ml. Tissue titer plots depict urine (**U**), bladder (**B**) and kidney (**K**) titers of individual mice at 4 wpi, grouped by outcome of longitudinal urinalysis: resolved bacteriuria (**R**) or persistent bacteriuria (**PB**). Solid lines connect the different urine and tissue titers from the same mouse. Dotted horizontal lines in tissue titer plots indicate the limits of detection. Data are combined from at least two independent experiments for each inbred mouse strain.
(0.67 MB DOC)

Figure S2 BALB/cJ and 129S1 mice are resistant to chronic cystitis with the UPEC strain, UTI89. Time course of bacteriuria and tissue titers were assayed after 4 weeks of infection with 10^7^ or 10^8^ cfu of either UTI89 Kan^R^ or UTI89 as indicated in Table 1, in *A*, BALB/cJ and *B*, 129S1/SvImJ mice. Solid lines connect the urine titers over time for each individual mouse. Dashed horizontal lines in time courses represent the cutoff for significant bacteriuria in free catch urines: 10^4^ cfu/ml. Tissue titer plots depict urine (**U**), bladder (**B**) and kidney (**K**) titers of individual mice at 4 wpi, grouped by outcome of longitudinal urinalysis: resolved bacteriuria (**R**) or persistent bacteriuria (**PB**). Solid lines connect the different urine and tissue titers from the same mouse. Dotted horizontal lines in tissue titer plots indicate the limits of detection. Data are combined from two independent experiments for each inbred mouse strain, except for BALB/cJ mice infected with 10^7^ cfu, which is from a single experiment.
(0.28 MB DOC)

Figure S3 Chronic cystitis in C3H/HeN mice is not restricted to infection with UTI89. Time course of bacteriuria and tissue titers in C3H/HeN mice were assayed after 4 weeks of infection with either 10^7^ or 10^8^ cfu of *A*, J96, a human UPEC isolate, or *B*, Top52, a *Klebsiella pneumonia* strain isolated from a woman with cystitis. Solid lines connect the urine titers over time for each individual mouse. Dashed horizontal lines in time courses represent the cutoff for significant bacteriuria in free catch urines: 10^4^ cfu/ml. Tissue titer plots depict urine (**U**), bladder (**B**) and kidney (**K**) titers of individual mice at 4 wpi, grouped by outcome of longitudinal urinalysis: resolved bacteriuria (**R**) or persistent bacteriuria (**PB**). Solid lines connect the different urine and tissue titers from the same mouse. Dotted horizontal lines in tissue titer plots indicate the limits of detection. Data in panel *A* are combined from two independent experiments and the data from panel *B* are from a single experiment.
(0.28 MB DOC)

Figure S4 Parameters of severe acute inflammation at 24 hpi are predictive of persistent bacteriuria with UPEC in C3H/HeOuJ, but not C3H/HeJ, mice. C3H/HeOuJ (closed circles) and C3H/HeJ (open circles) mice were infected with 10^7^ cfu UTI89 Kan^R^ and assessed at 24 hpi for *A*, pyuria, *B*, weight loss and *C*, serum cytokine levels. Mice were grouped by the outcome of longitudinal urinalysis over 4 wpi, i.e. whether they resolved bacteriuria (**R**), were persistently bacteriuric (**PB**), or in the case of C3H/HeJ mice whether they died during the course of infection (**D**). Data are combined from two independent experiments. All statistics are by Mann-Whitney U two-tailed test: *, *P*<0.05, **, *P*<0.01, ***, *P*<0.001 and **ns**, not significant; horizontal bars indicate median values.
(0.27 MB DOC)

Figure S5 Serum cytokines are also predictive of the development of chronic cystitis in C3H/HeN mice after infection with the UPEC strain, J96. C3H/HeN mice were infected with either 10^7^ or 10^8^ cfu of the UPEC strain J96 and sera were collected at 24 hpi for cytokine analysis. Mice were grouped by the outcome of longitudinal urinalysis over 4 wpi, i.e. whether they resolved bacteriuria (**R**) or were persistently bacteriuric (**PB**). Data from both inoculums in two independent experiments are combined in the analysis. All statistics are by Mann-Whitney U two-tailed test: *, *P*<0.05; horizontal bars indicate median values.
(0.30 MB DOC)

Figure S6 Urine cytokines are also predictive of the development of chronic cystitis. C3H/HeN mice were infected with 10^7^ cfu UTI89 Kan^R^ and urines were collected at 24 hpi for cytokine analysis. Mice were grouped by the outcome of longitudinal urinalysis over 4 wpi, i.e. whether they resolved bacteriuria (**R**) or were persistently bacteriuric (**PB**), with a PBS-infected group (**Mock**) as controls. Data from two independent experiments are combined in the analysis. One outlier mouse from the resolved group was excluded from the analysis because she had a renal abscess, an outcome not previously observed in C3H/HeN mice. All statistics are by Mann-Whitney U two-tailed test: *, *P*<0.05, **, *P*<0.01 and **ns**, not significant with *P*>0.10 unless otherwise indicated; horizontal bars indicate median values.
(0.32 MB DOC)

Figure S7 C3H*scid* mice resolved UPEC infection more readily than their congenic strain, C3H/HeSnJ, and have less acute weight loss during acute infection. C3H/HeSnJ (closed circles) and C3Smn.CB17-*Prkdc^scid^*/J (open circles) mice were infected with 10^8^ cfu of either UTI89 Kan^R^ or UTI89. Data are combined from 4 independent experiments. *A*, The time course of bacteriuria over 4 wpi was determined by longitudinal urinalysis. Solid lines connect the urine titers over time for each individual mouse. Horizontal dashed lines represent the cutoff for significant bacteriuria in free catch urines: 10^4^ cfu/ml. *B*, Acute weight loss was assessed at 24 hpi. Statistics are by Mann-Whitney U two-tailed test: ***, *P*<0.001; horizontal bars indicate median values.
(0.36 MB DOC)

Figure S8 Dexamethasone pre-treatment of C3H/HeN mice protects against chronic infection, despite similar bladder bacterial burdens at 24 hpi. C3H/HeN mice were infected with 10^8^ cfu UTI89 Kan^R^ 2 hours after pre-treatment by intraperitoneal injection with either 200ug dexamethasone sodium phosphate (open circles) diluted in sterile saline or sterile saline alone (closed circles). *A*, the time course of bacteriuria over 4 wpi was determined by longitudinal urinalysis and the data from three independent experiments combined. Solid lines connect the urine titers over time for each individual mouse. Horizontal dashed lines represent the cutoff for significant bacteriuria in free catch urines: 10^4^ cfu/ml. *B*, mice were sacrificed at 24 hpi and bladder bacterial burdens assayed. Statistics are by Mann-Whitney U two-tailed test: **ns**, not significant; horizontal bars indicate median values.
(0.27 MB DOC)
