# Supplementary material for: Early Severe Inflammatory Responses to Uropathogenic E. coli Predispose to Chronic and Recurrent Urinary Tract Infection
Source: PLoS Pathog. 2010 Aug 12;6(8):e1001042. doi: 10.1371/journal.ppat.1001042 (PMC2930321; doi:10.1371/journal.ppat.1001042)
Supplement: Figure S1 — Numerous C3H and related inbred mouse strains are susceptible to chronic cystitis with the UPEC strain, UTI89. Time course of bacteriuria and tissue titers were assayed after 4 weeks of infection with 107 or 108 cfu of either UTI89 KanR or UTI89 as indicated in Table 1, in A, C3H/HeSnJ, B, C3HeB/FeJ, C, CBA/J and D, DBA/2J mice. Solid lines connect the urine titers over time for each individual mouse. Dashed horizontal lines in time courses represent the cutoff for significant bacteriuria in free catch urines: 104 cfu/ml. Tissue titer plots depict urine (U), bladder (B) and kidney (K) titers of individual mice at 4 wpi, grouped by outcome of longitudinal urinalysis: resolved bacteriuria (R) or persistent bacteriuria (PB). Solid lines connect the different urine and tissue titers from the same mouse. Dotted horizontal lines in tissue titer plots indicate the limits of detection. Data are combined from at least two independent experiments for each inbred mouse strain. (0.67 MB DOC) [file ppat.1001042.s001.doc]

**
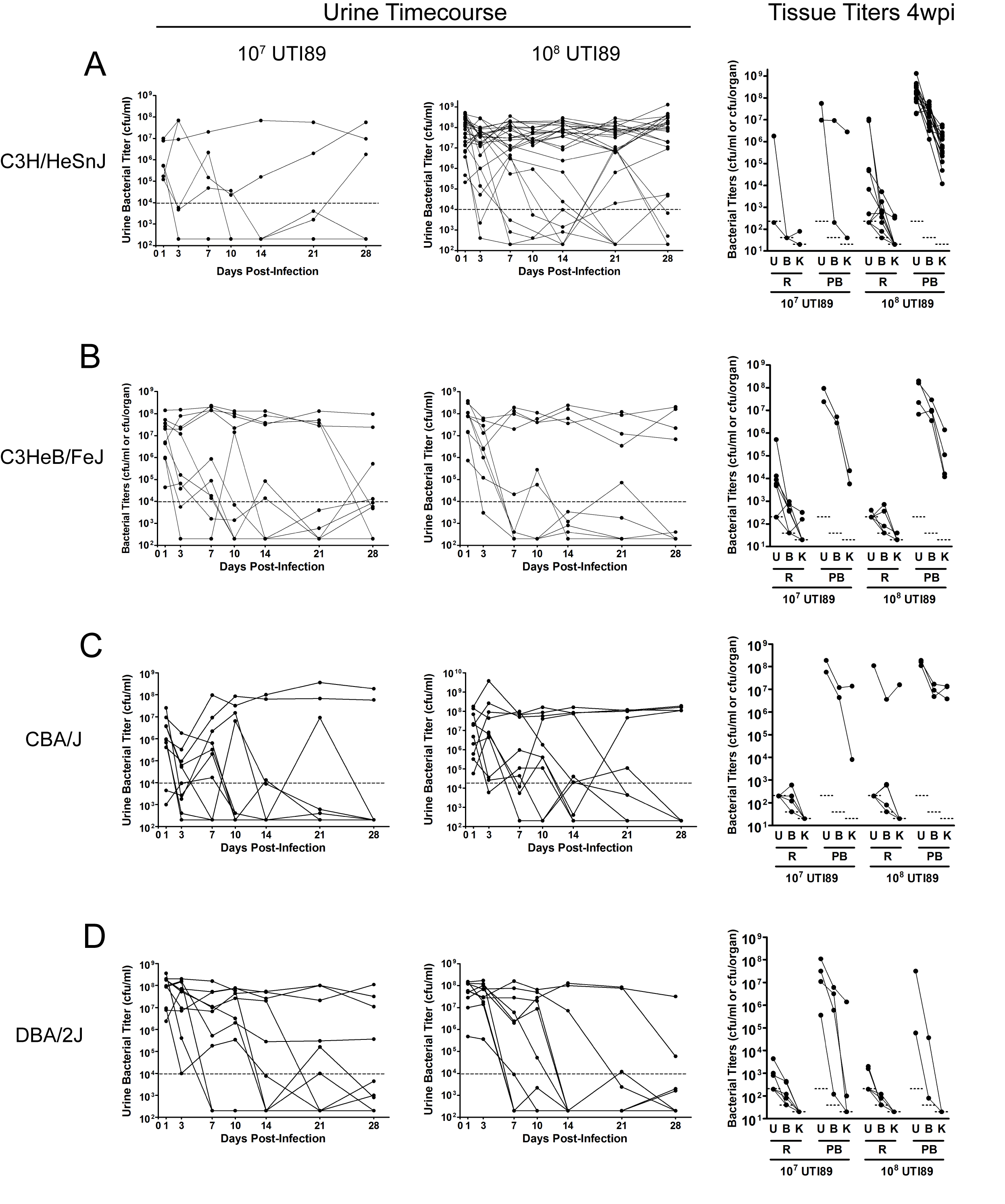
**

**Figure S1. Numerous C3H and related inbred mouse strains are susceptible to chronic cystitis with the UPEC strain, UTI89.** Time course of bacteriuria and tissue titers were assayed after 4 weeks of infection with 10^7^ or 10^8^ cfu of either UTI89 Kan^R^ or UTI89 as indicated in Table 1, in *A*, C3H/HeSnJ, *B*, C3HeB/FeJ, *C*, CBA/J and *D*, DBA/2J mice. Solid lines connect the urine titers over time for each individual mouse. Dashed horizontal lines in time courses represent the cutoff for significant bacteriuria in free catch urines: 10^4^ cfu/ml. Tissue titer plots depict urine (**U**), bladder (**B**) and kidney (**K**) titers of individual mice at 4 wpi, grouped by outcome of longitudinal urinalysis: resolved bacteriuria (**R**) or persistent bacteriuria (**PB**). Solid lines connect the different urine and tissue titers from the same mouse. Dotted horizontal lines in tissue titer plots indicate the limits of detection. Data are combined from at least two independent experiments for each inbred mouse strain.
