# Supplementary material for: Early Severe Inflammatory Responses to Uropathogenic E. coli Predispose to Chronic and Recurrent Urinary Tract Infection
Source: PLoS Pathog. 2010 Aug 12;6(8):e1001042. doi: 10.1371/journal.ppat.1001042 (PMC2930321; doi:10.1371/journal.ppat.1001042)
Supplement: Figure S3 — Chronic cystitis in C3H/HeN mice is not restricted to infection with UTI89. Time course of bacteriuria and tissue titers in C3H/HeN mice were assayed after 4 weeks of infection with either 107 or 108 cfu of A, J96, a human UPEC isolate, or B, Top52, a Klebsiella pneumonia strain isolated from a woman with cystitis. Solid lines connect the urine titers over time for each individual mouse. Dashed horizontal lines in time courses represent the cutoff for significant bacteriuria in free catch urines: 104 cfu/ml. Tissue titer plots depict urine (U), bladder (B) and kidney (K) titers of individual mice at 4 wpi, grouped by outcome of longitudinal urinalysis: resolved bacteriuria (R) or persistent bacteriuria (PB). Solid lines connect the different urine and tissue titers from the same mouse. Dotted horizontal lines in tissue titer plots indicate the limits of detection. Data in panel A are combined from two independent experiments and the data from panel B are from a single experiment. (0.28 MB DOC) [file ppat.1001042.s003.doc]

**
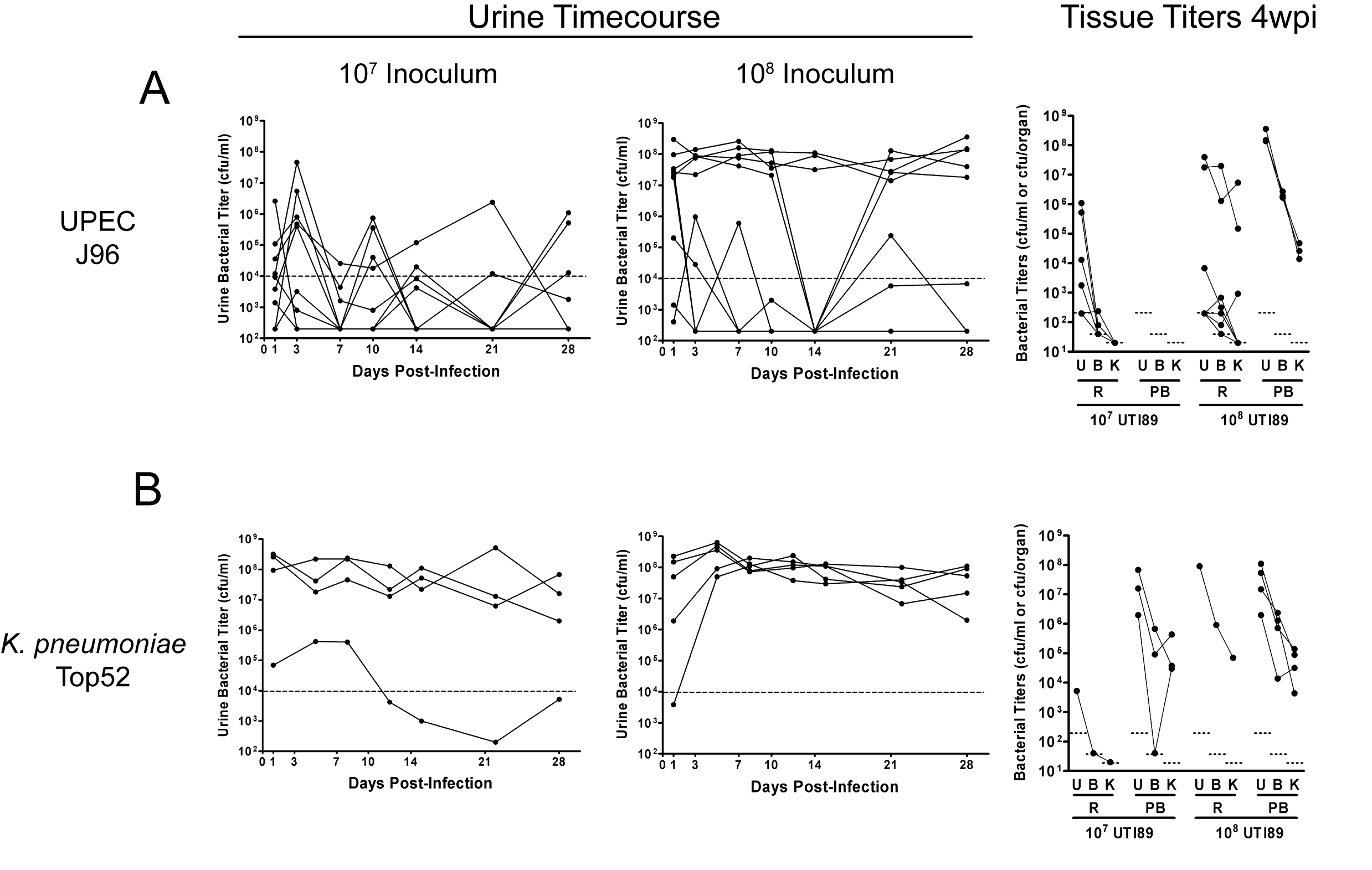
**

**Figure S3. Chronic cystitis in C3H/HeN mice is not restricted to infection with UTI89.** Time course of bacteriuria and tissue titers in C3H/HeN mice were assayed after 4 weeks of infection with either 10^7^ or 10^8^ cfu of *A*, J96, a human UPEC isolate, or *B*, Top52, a *Klebsiella pneumonia* strain isolated from a woman with cystitis. Solid lines connect the urine titers over time for each individual mouse. Dashed horizontal lines in time courses represent the cutoff for significant bacteriuria in free catch urines: 10^4^ cfu/ml. Tissue titer plots depict urine (**U**), bladder (**B**) and kidney (**K**) titers of individual mice at 4 wpi, grouped by outcome of longitudinal urinalysis: resolved bacteriuria (**R**) or persistent bacteriuria (**PB**). Solid lines connect the different urine and tissue titers from the same mouse. Dotted horizontal lines in tissue titer plots indicate the limits of detection. Data in panel *A* are combined from two independent experiments and the data from panel *B* are from a single experiment.
