# Supplementary material for: Early Severe Inflammatory Responses to Uropathogenic E. coli Predispose to Chronic and Recurrent Urinary Tract Infection
Source: PLoS Pathog. 2010 Aug 12;6(8):e1001042. doi: 10.1371/journal.ppat.1001042 (PMC2930321; doi:10.1371/journal.ppat.1001042)
Supplement: Figure S4 — Parameters of severe acute inflammation at 24 hpi are predictive of persistent bacteriuria with UPEC in C3H/HeOuJ, but not C3H/HeJ, mice. C3H/HeOuJ (closed circles) and C3H/HeJ (open circles) mice were infected with 107 cfu UTI89 KanR and assessed at 24 hpi for A, pyuria, B, weight loss and C, serum cytokine levels. Mice were grouped by the outcome of longitudinal urinalysis over 4 wpi, i.e. whether they resolved bacteriuria (R), were persistently bacteriuric (PB), or in the case of C3H/HeJ mice whether they died during the course of infection (D). Data are combined from two independent experiments. All statistics are by Mann-Whitney U two-tailed test: *, P<0.05, **, P<0.01, ***, P<0.001 and ns, not significant; horizontal bars indicate median values. (0.27 MB DOC) [file ppat.1001042.s004.doc]

**
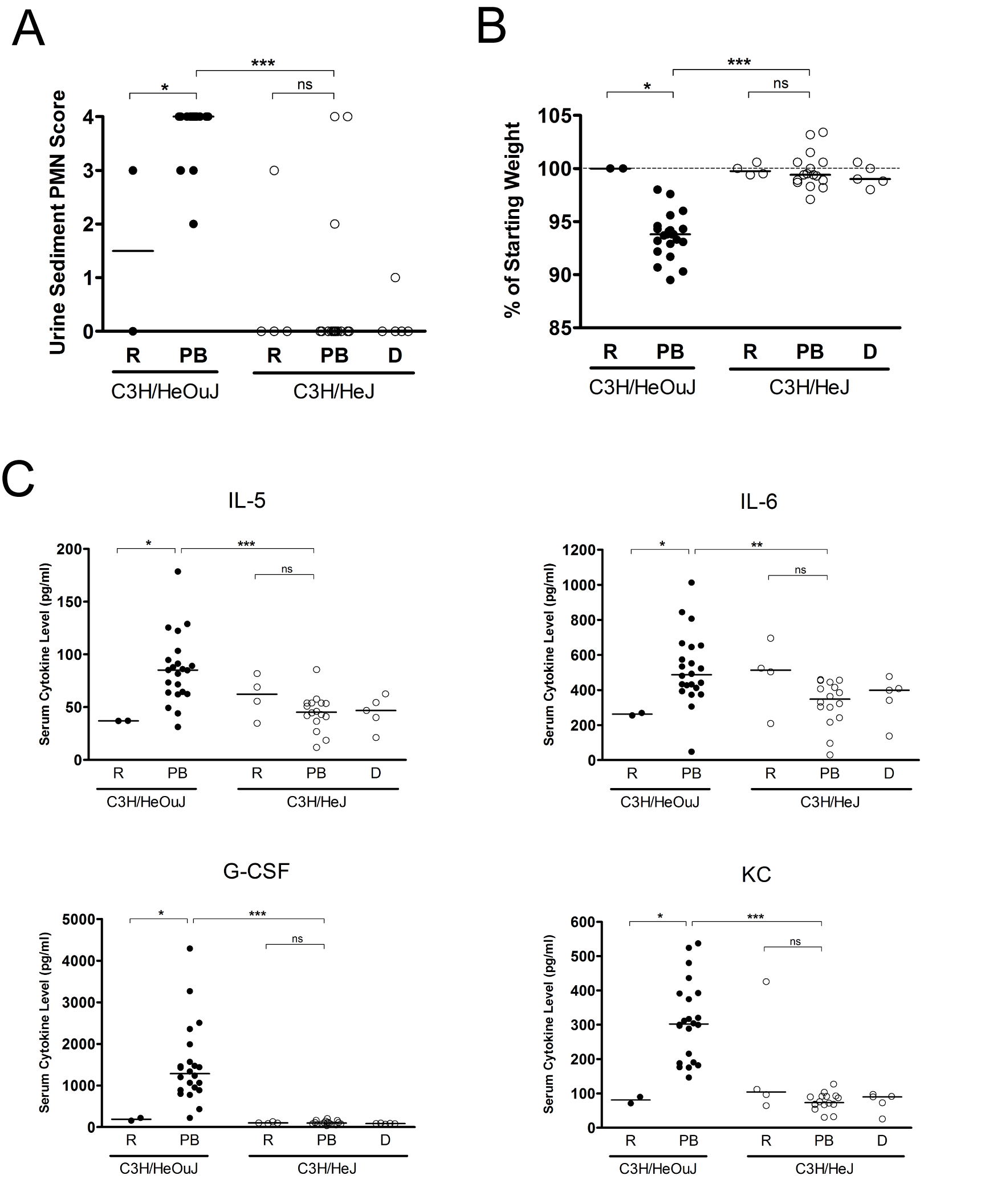
**

**Figure S4. Parameters of severe acute inflammation at 24 hpi are predictive of persistent bacteriuria with UPEC in C3H/HeOuJ, but not C3H/HeJ, mice.** C3H/HeOuJ (closed circles) and C3H/HeJ (open circles) mice were infected with 10^7^ cfu UTI89 Kan^R^ and assessed at 24 hpi for *A*, pyuria, *B*, weight loss and *C*, serum cytokine levels. Mice were grouped by the outcome of longitudinal urinalysis over 4 wpi, i.e. whether they resolved bacteriuria (**R**), were persistently bacteriuric (**PB**), or in the case of C3H/HeJ mice whether they died during the course of infection (**D**). Data are combined from two independent experiments. All statistics are by Mann-Whitney U two-tailed test: *****, *P* < 0.05, ******, *P* < 0.01, *******, *P* < 0.001 and **ns**, not significant; horizontal bars indicate median values.
