# Supplementary material for: Early Severe Inflammatory Responses to Uropathogenic E. coli Predispose to Chronic and Recurrent Urinary Tract Infection
Source: PLoS Pathog. 2010 Aug 12;6(8):e1001042. doi: 10.1371/journal.ppat.1001042 (PMC2930321; doi:10.1371/journal.ppat.1001042)
Supplement: Figure S5 — Serum cytokines are also predictive of the development of chronic cystitis in C3H/HeN mice after infection with the UPEC strain, J96. C3H/HeN mice were infected with either 107 or 108 cfu of the UPEC strain J96 and sera were collected at 24 hpi for cytokine analysis. Mice were grouped by the outcome of longitudinal urinalysis over 4 wpi, i.e. whether they resolved bacteriuria (R) or were persistently bacteriuric (PB). Data from both inoculums in two independent experiments are combined in the analysis. All statistics are by Mann-Whitney U two-tailed test: *, P<0.05; horizontal bars indicate median values. (0.30 MB DOC) [file ppat.1001042.s005.doc]

**
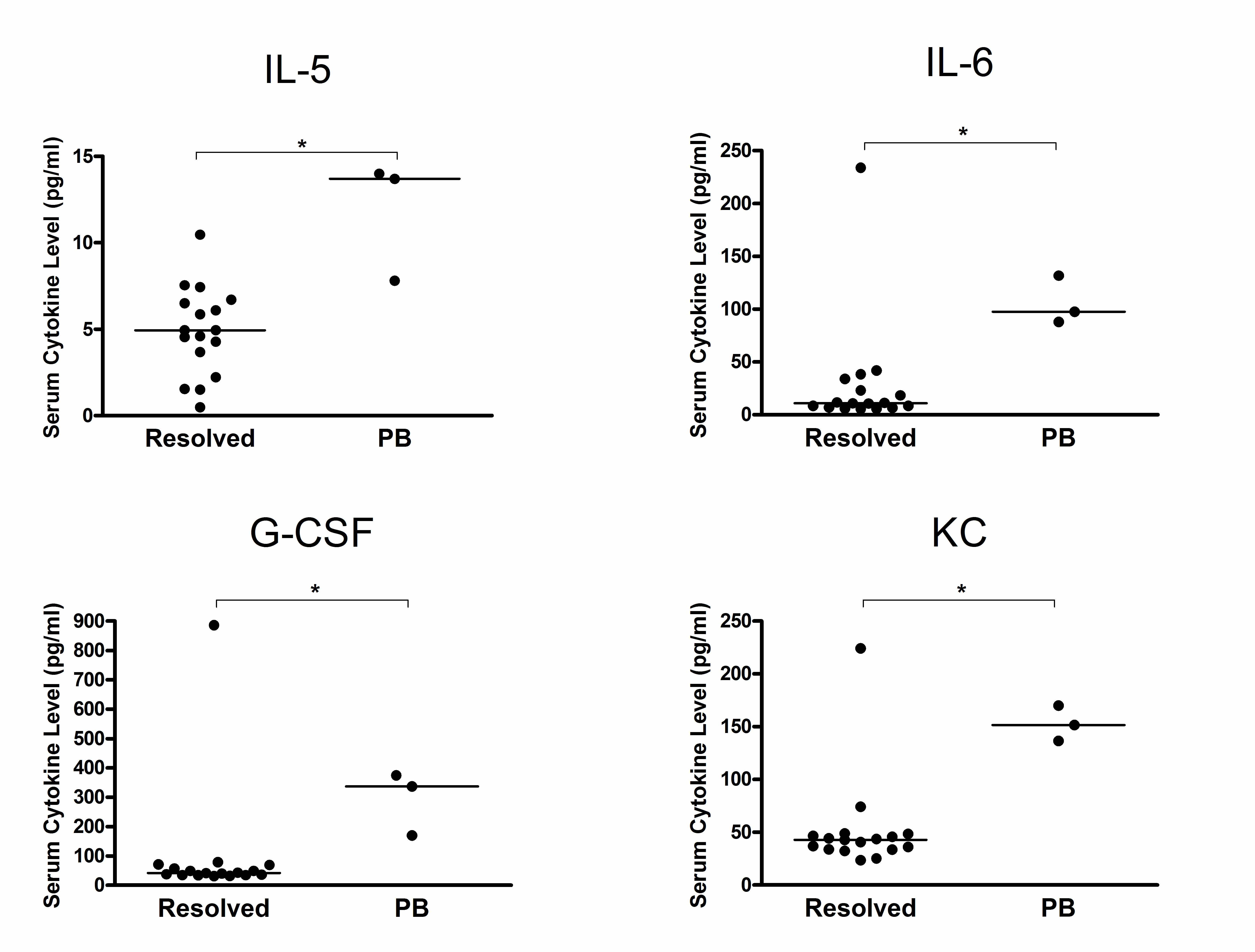
**

**Figure S5. Serum cytokines are also predictive of the development of chronic cystitis in C3H/HeN mice after infection with the UPEC strain, J96.** C3H/HeN mice were infected with either 10^7^ or 10^8^ cfu of the UPEC strain J96 and sera were collected at 24 hpi for cytokine analysis. Mice were grouped by the outcome of longitudinal urinalysis over 4 wpi, i.e. whether they resolved bacteriuria (**R**) or were persistently bacteriuric (**PB**). Data from both inoculums in two independent experiments are combined in the analysis. All statistics are by Mann-Whitney U two-tailed test: *****, *P* < 0.05; horizontal bars indicate median values.
