# Supplementary material for: Early Severe Inflammatory Responses to Uropathogenic E. coli Predispose to Chronic and Recurrent Urinary Tract Infection
Source: PLoS Pathog. 2010 Aug 12;6(8):e1001042. doi: 10.1371/journal.ppat.1001042 (PMC2930321; doi:10.1371/journal.ppat.1001042)
Supplement: Figure S6 — Urine cytokines are also predictive of the development of chronic cystitis. C3H/HeN mice were infected with 107 cfu UTI89 KanR and urines were collected at 24 hpi for cytokine analysis. Mice were grouped by the outcome of longitudinal urinalysis over 4 wpi, i.e. whether they resolved bacteriuria (R) or were persistently bacteriuric (PB), with a PBS-infected group (Mock) as controls. Data from two independent experiments are combined in the analysis. One outlier mouse from the resolved group was excluded from the analysis because she had a renal abscess, an outcome not previously observed in C3H/HeN mice. All statistics are by Mann-Whitney U two-tailed test: *, P<0.05, **, P<0.01 and ns, not significant with P>0.10 unless otherwise indicated; horizontal bars indicate median values. (0.32 MB DOC) [file ppat.1001042.s006.doc]

**
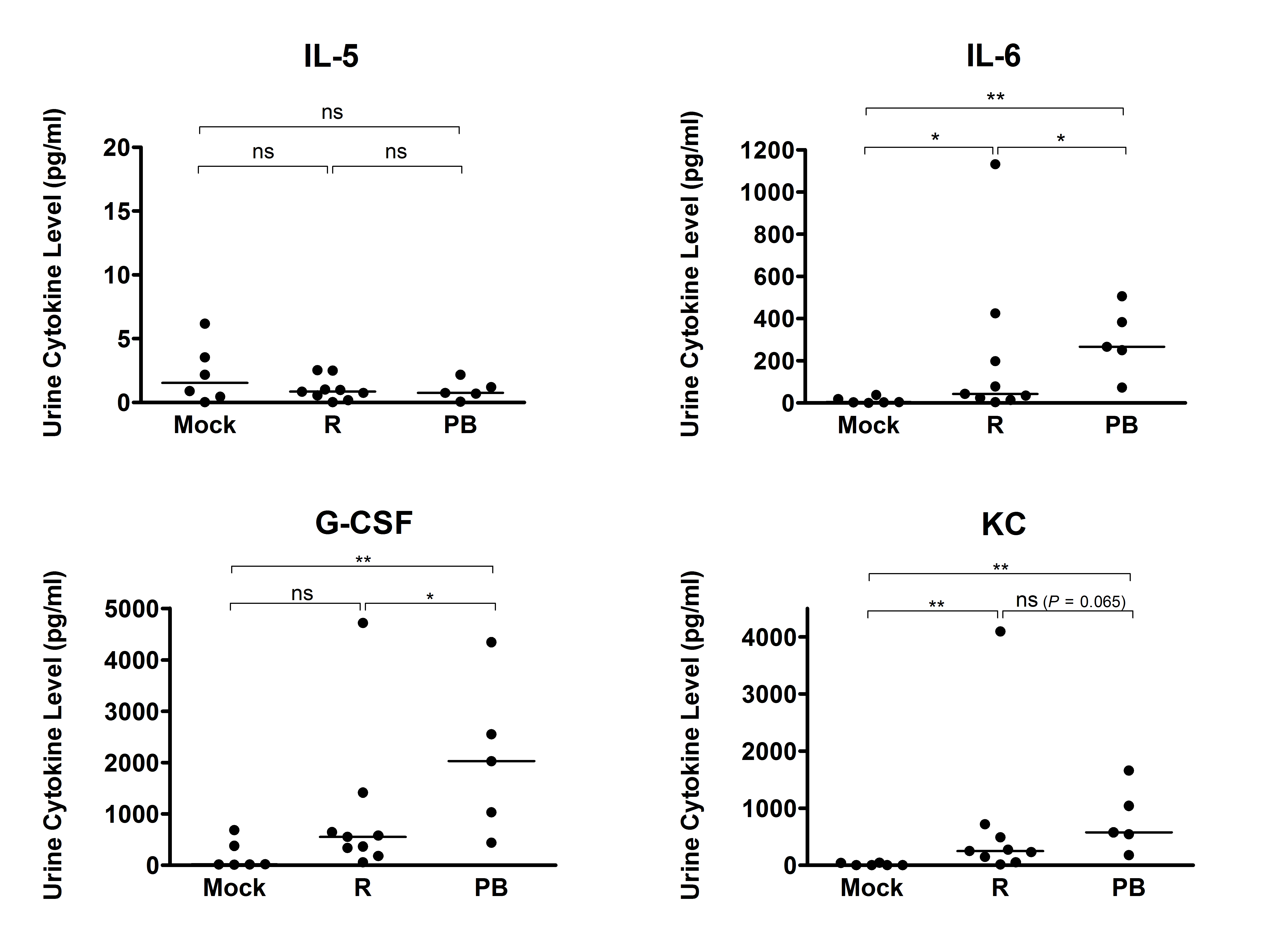
**

**Figure S6. Urine cytokines are also predictive of the development of chronic cystitis.** C3H/HeN mice were infected with 10^7^ cfu UTI89 Kan^R^ and urines were collected at 24 hpi for cytokine analysis. Mice were grouped by the outcome of longitudinal urinalysis over 4 wpi, i.e. whether they resolved bacteriuria (**R**) or were persistently bacteriuric (**PB**), with a PBS-infected group (**Mock**) as controls. Data from two independent experiments are combined in the analysis. One outlier mouse from the resolved group was excluded from the analysis because she had a renal abscess, an outcome not previously observed in C3H/HeN mice. All statistics are by Mann-Whitney U two-tailed test: *****, *P* < 0.05, ******, *P* < 0.01 and **ns**, not significant with *P* > 0.10 unless otherwise indicated; horizontal bars indicate median values.
