# Supplementary material for: Early Severe Inflammatory Responses to Uropathogenic E. coli Predispose to Chronic and Recurrent Urinary Tract Infection
Source: PLoS Pathog. 2010 Aug 12;6(8):e1001042. doi: 10.1371/journal.ppat.1001042 (PMC2930321; doi:10.1371/journal.ppat.1001042)
Supplement: Figure S7 — C3Hscid mice resolved UPEC infection more readily than their congenic strain, C3H/HeSnJ, and have less acute weight loss during acute infection. C3H/HeSnJ (closed circles) and C3Smn.CB17-Prkdcscid/J (open circles) mice were infected with 108 cfu of either UTI89 KanR or UTI89. Data are combined from 4 independent experiments. A, The time course of bacteriuria over 4 wpi was determined by longitudinal urinalysis. Solid lines connect the urine titers over time for each individual mouse. Horizontal dashed lines represent the cutoff for significant bacteriuria in free catch urines: 104 cfu/ml. B, Acute weight loss was assessed at 24 hpi. Statistics are by Mann-Whitney U two-tailed test: ***, P<0.001; horizontal bars indicate median values. (0.36 MB DOC) [file ppat.1001042.s007.doc]

**
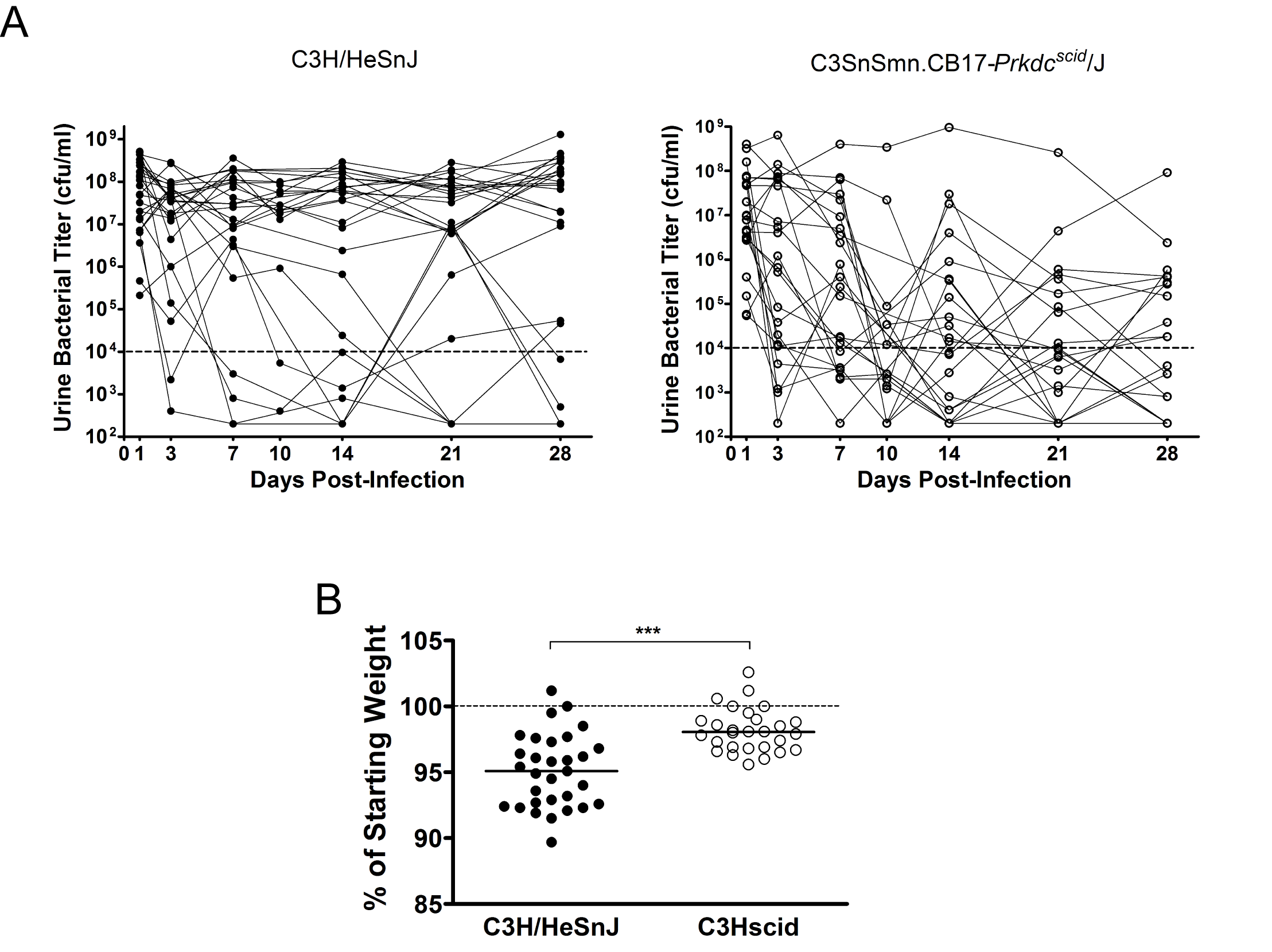
**

**Figure S7. C3H*scid* mice resolved UPEC infection more readily than their congenic strain, C3H/HeSnJ, and have less acute weight loss during acute infection.** C3H/HeSnJ (closed circles) and C3Smn.CB17-*Prkdc^scid^*/J (open circles) mice were infected with 10^8^ cfu of either UTI89 Kan^R^ or UTI89. Data are combined from 4 independent experiments. *A*, The time course of bacteriuria over 4 wpi was determined by longitudinal urinalysis. Solid lines connect the urine titers over time for each individual mouse. Horizontal dashed lines represent the cutoff for significant bacteriuria in free catch urines: 10^4^ cfu/ml. *B*, Acute weight loss was assessed at 24 hpi. Statistics are by Mann-Whitney U two-tailed test: *******, *P* < 0.001; horizontal bars indicate median values.
