# Supplementary material for: Early Severe Inflammatory Responses to Uropathogenic E. coli Predispose to Chronic and Recurrent Urinary Tract Infection
Source: PLoS Pathog. 2010 Aug 12;6(8):e1001042. doi: 10.1371/journal.ppat.1001042 (PMC2930321; doi:10.1371/journal.ppat.1001042)
Supplement: Figure S8 — Dexamethasone pre-treatment of C3H/HeN mice protects against chronic infection, despite similar bladder bacterial burdens at 24 hpi. C3H/HeN mice were infected with 108 cfu UTI89 KanR 2 hours after pre-treatment by intraperitoneal injection with either 200ug dexamethasone sodium phosphate (open circles) diluted in sterile saline or sterile saline alone (closed circles). A, the time course of bacteriuria over 4 wpi was determined by longitudinal urinalysis and the data from three independent experiments combined. Solid lines connect the urine titers over time for each individual mouse. Horizontal dashed lines represent the cutoff for significant bacteriuria in free catch urines: 104 cfu/ml. B, mice were sacrificed at 24 hpi and bladder bacterial burdens assayed. Statistics are by Mann-Whitney U two-tailed test: ns, not significant; horizontal bars indicate median values. (0.27 MB DOC) [file ppat.1001042.s008.doc]

**
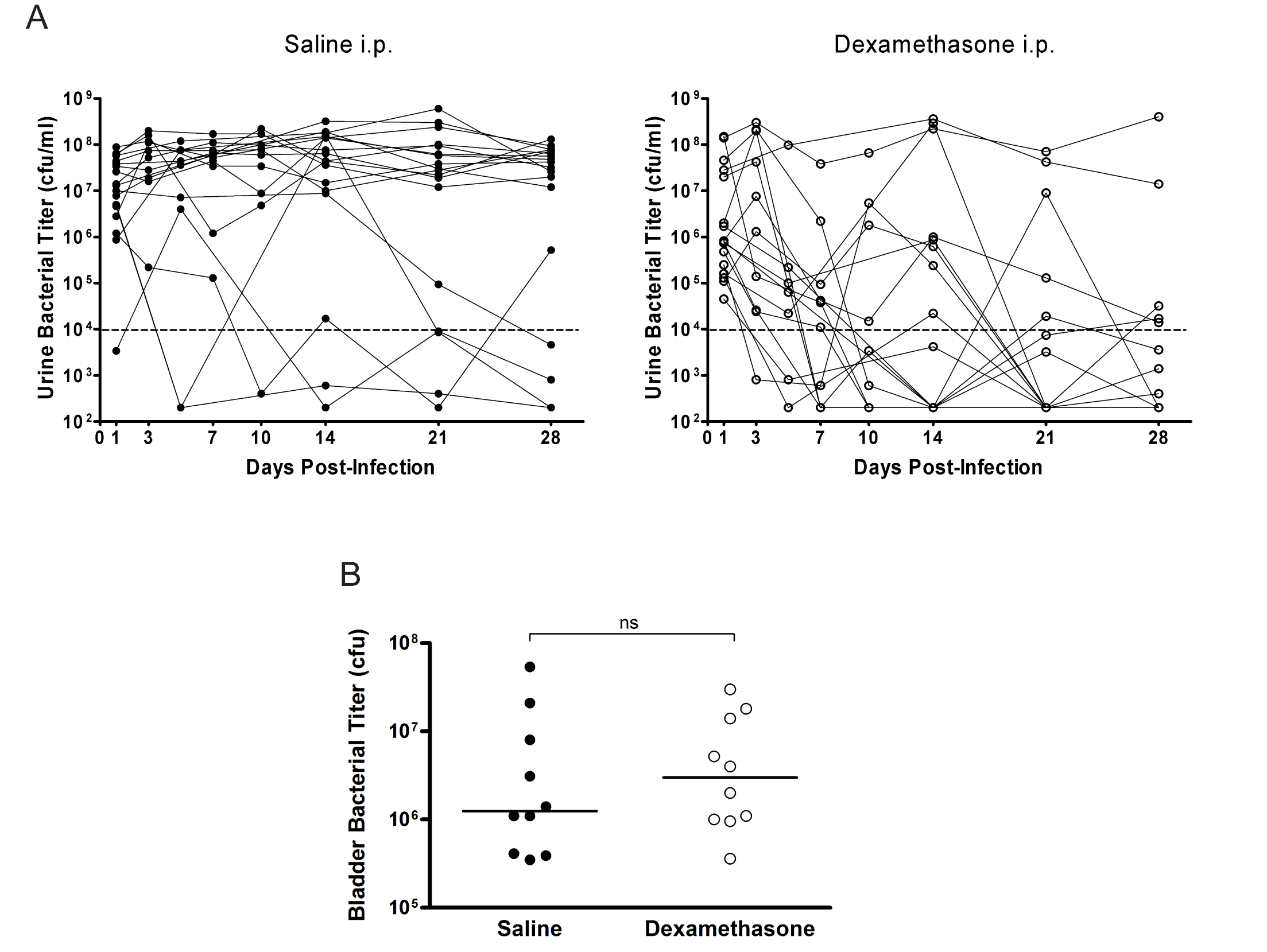
**

**Figure S8. Dexamethasone pre-treatment of C3H/HeN mice protects against chronic infection, despite similar bladder bacterial burdens at 24 hpi.** C3H/HeN mice were infected with 10^8^ cfu UTI89 Kan^R^ 2 hours after pre-treatment by intraperitoneal injection with either 200ug dexamethasone sodium phosphate (open circles) diluted in sterile saline or sterile saline alone (closed circles). *A*, the time course of bacteriuria over 4 wpi was determined by longitudinal urinalysis and the data from three independent experiments combined. Solid lines connect the urine titers over time for each individual mouse. Horizontal dashed lines represent the cutoff for significant bacteriuria in free catch urines: 10^4^ cfu/ml. *B*, mice were sacrificed at 24 hpi and bladder bacterial burdens assayed. Statistics are by Mann-Whitney U two-tailed test: **ns**, not significant; horizontal bars indicate median values.
